# Mapping Neuroimaging Findings of Creativity and Brain Disease Onto a Common Brain Circuit

**DOI:** 10.1001/jamanetworkopen.2024.59297

**Published:** 2025-02-13

**Authors:** Julian Kutsche, Joseph J. Taylor, Michael G. Erkkinen, Haya Akkad, Sanaz Khosravani, William Drew, Anna Abraham, Derek V. M. Ott, Juliana Wall, Alexander Li Cohen, Andreas Horn, Wolf-Julian Neumann, Isaiah Kletenik, Michael D. Fox

**Affiliations:** 1 Department of Neurology and Experimental Neurology, Charité – Universitätsmedizin Berlin, Berlin, Germany; 2 Center for Brain Circuit Therapeutics, Brigham and Women’s Hospital, Harvard Medical School, Harvard University, Boston, Massachusetts; 3 Department of Neurology, Brigham and Women’s Hospital, Harvard Medical School, Harvard University, Boston, Massachusetts; 4 Department of Psychiatry, Brigham and Women’s Hospital, Harvard Medical School, Harvard University, Boston, Massachusetts; 5 Institute of Cognitive Neuroscience, University College London, London, United Kingdom; 6 Department of Educational Psychology, Mary Frances Early College of Education, University of Georgia, Athens; 7 Department of Neurology, Max Planck Institute for Human Cognitive and Brain Sciences, Leipzig, Germany; 8 Department of Neurology, Boston Children’s Hospital, Harvard Medical School, Harvard University, Boston, Massachusetts; 9 Department of Radiology, Brigham and Women’s Hospital, Harvard Medical School, Harvard University, Boston, Massachusetts

## Abstract

**Importance:**

Creativity is important for problem solving, adaptation to a changing environment, and innovation. Neuroimaging studies seeking to map creativity have yielded conflicting results, and studies of patients with brain disease have reported both decreases and paradoxical increases in creativity, leaving the neural basis of creativity unclear.

**Objective:**

To investigate the brain circuit underlying creativity and assess its association with brain injury and neurodegenerative disease.

**Design, Setting, and Participants:**

This study examined neuroimaging coordinates from a meta-analysis of 36 studies published between 2004 and 2019 associated with increased activity during creative tasks in healthy participants. A validated method termed *coordinate network mapping* and a database of resting-state functional connectivity from 1000 healthy individuals were used to test whether these coordinates mapped to a common brain circuit. Specificity was assessed through comparison to random coordinates and coordinates from working memory tasks in healthy participants. Reproducibility was assessed using an independent dataset of coordinates from additional studies of creativity in healthy participants. Finally, alignment with effects of focal brain damage on creativity was tested using data from patients with brain lesions and coordinates of brain atrophy from 7 different neurodegenerative disorders.

**Main Outcomes and Measures:**

The primary outcomes were creativity or no creativity and alignment with a creativity circuit or no alignment.

**Results:**

Creativity tasks activated heterogenous locations, with coordinates scattered across many different brain regions (415 coordinates derived from 857 healthy participants; pooled mean [SD] age, 24.1 [6.91] years; 461 [54%] female). However, these activation coordinates were part of a common brain circuit, defined by negative connectivity to the right frontal pole. This result was consistent across creative domains, reproducible in an independent dataset (383 coordinates derived from 691 participants) and specific to creativity when compared with random gray matter coordinates (n = 415) or coordinates activated by working memory tasks (3072 coordinates derived from 2900 healthy participants). Damage to this creativity circuit by lesions (n = 56 patients) or neurodegenerative disease (2262 coordinates derived from 4804 patients) aligned with both decreases and increases in creativity observed in these disorders.

**Conclusions and Relevance:**

Findings from this study suggest that brain regions activated by creativity tasks map to a brain circuit defined by negative functional connectivity to the right frontal pole. Damage to this circuit aligned with changes in creativity observed in individuals with certain brain diseases, including paradoxical creativity increases.

## Key Points

**Question:**

Does creativity map to a specific brain circuit, and does damage to this circuit align with creativity changes that occur in brain disease?

**Findings:**

This study using network mapping of meta-analytic data involving 857 participants found that brain regions activated by creativity tasks mapped to a human brain circuit centered on the right frontal pole. Damage to this circuit aligned with both decreases and paradoxical increases in creativity observed across multiple different brain diseases.

**Meaning:**

Findings from this study suggest that creativity maps to a specific brain circuit in healthy individuals and that damage to this circuit in individuals with certain neurodegenerative diseases may explain their paradoxical increases in creativity; further research is needed to confirm and validate these findings.

## Teaser

This study using network mapping of meta-analytic data investigates whether creativity maps to a specific brain circuit and whether damage to that circuit aligns with creativity changes observed in persons with brain disease.

## Introduction

Creativity involves generation of novel and useful products or ideas in a changing environment.^1^ It is important for problem solving and is the foundation for technological, artistic, and cultural innovation. The neuroanatomical substrate of creativity has been a topic of long-standing neuroscientific interest^2^ but may also be relevant for medical practitioners in understanding symptoms among patients with brain disease.^3^ Some evidence suggests creativity can be affected by brain disease, including focal brain damage^4,5,6^ and neurodegenerative disorders, as well as by treatments for brain disease.^7,8,9,10^ Brain disease can lead to decreases or paradoxical increases in creativity.^8,11,12,13,14,15,16^ Despite long-standing interest in creativity and its relationship to brain disease,^17^ it remains unclear which brain regions are most important for creativity.

The neural underpinnings of creativity have been studied in 2 main ways: (1) neuroimaging studies in healthy participants and (2) alterations in creativity in patients with brain disease. Both approaches have identified brain regions involved in creativity, but results have been heterogenous across studies. Among healthy participants, different creative tasks tend to activate different brain regions, suggesting that different types of creativity may map to different neuroanatomy.^18,19,20,21^ However, these results do not preclude the possibility of a domain-general neuroanatomical substrate for creativity, the existence of which remains an ongoing topic of debate.^22,23^ Other neuroimaging studies have examined the relationship between creativity and brain network connectivity.^24,25,26,27,28,29,30^ These articles suggest that creativity may map better to brain networks than individual brain regions, but exactly which network and how these networks relate to brain regions activated by creativity tasks remains unclear.

Among patients, different brain regions have been implicated across different disorders.^4,31,32,33^ For example, creativity increases have been observed in patients with semantic variant primary progressive aphasia (svPPA),^12^ which is associated with atrophy to the anterior lateral temporal lobes. However, associations of this brain region with creativity are not consistent,^34^ and other brain regions, such as the dorsolateral prefrontal cortex, have been associated with facilitation of creativity in patients with neurodegenerative disease.^35,36^

The aim of this study was to take a network approach to localizing creativity in the brain across different creative domains, including music, writing, drawing, and divergent thinking. This network mapping approach can test whether heterogenous results observed across different studies map to a connected brain circuit.^37^ The approach has been validated for use with neuroimaging coordinates activated by different tasks,^38,39^ lesion locations,^40,41,42,43^ and coordinates of brain atrophy associated with neurodegeneration.^44^ It thus has the potential to identify convergent, domain-general neurocircuitry for creativity.

## Methods

This study using network mapping of meta-analytic data followed the Preferred Reporting Items for Systematic Reviews and Meta-analyses (PRISMA) reporting guideline. A full description of methods is given in the eMethods in Supplement 1. In brief, we extracted brain coordinates activated by creativity tasks (vs control tasks) from 36 prior functional magnetic resonance imaging (fMRI) studies published between 2004 and 2019 that were included in a meta-analysis.^20^

### Statistical Analysis

A validated method termed *coordinate network mapping* and resting-state functional connectivity data from 1000 heathy participants were used to identify the brain circuit functionally connected to each set of coordinates, resulting in 36 network connectivity maps.^38,39,45^ To identify brain regions common to these 36 maps, we thresholded the network maps (*t* ≥ 5), binarized them, then overlaid them. To identify brain regions that were statistically consistent across these 36 maps, we performed a 2-tailed 1-sample *t* test and identified significant clusters after correction for multiple comparisons (family-wise error rate, *P* < .05). To identify brain regions that were specific to these 36 network maps, we performed a 2-sample *t* test comparing the 36 maps derived from studies of creativity to (1) 36 network maps derived from random gray matter coordinates and (2) 150 network maps derived from fMRI studies of working memory. We again identified significant clusters after correction for multiple comparisons (false discovery rate *P* < .05). We used a conjunction analysis of the above results to identify any brain network connections that were both sensitive and specific for creativity. We validated the results by comparing them to coordinates activated by creativity tasks in an independent set of fMRI experiments (n = 30 studies) (eTable 3 in Supplement 1),^18,19^ the location of brain lesions associated with changes in performance on creativity tasks (n = 56 patients with lesions),^31^ and the locations of brain atrophy in patients with neurodegenerative disease associated with changes in creativity (n = 189 studies^46,47,48,49,50,51^) (eTable 5 in Supplement 1).

The functional connectivity data equivalent to those used in this study are available online through the Harvard Dataverse.^52^ The fMRI and atrophy data used in this study are publicly available and obtained from published medical literature (eTables 1, 3, 4 and 5 in Supplement 1). The threshold for statistical significance was a 2-tailed *P* < .05. Statistical neuroimaging analyses were performed in Matlab, version 2022b (Mathworks Inc) and using FSL software (version 5.0.10). Brain images were created using MRicroGL (version 12.7.6), FSLeyes (version 1.4.6), and Surf Ice (version 12.7.6) software.

## Results

**Figure 1.**
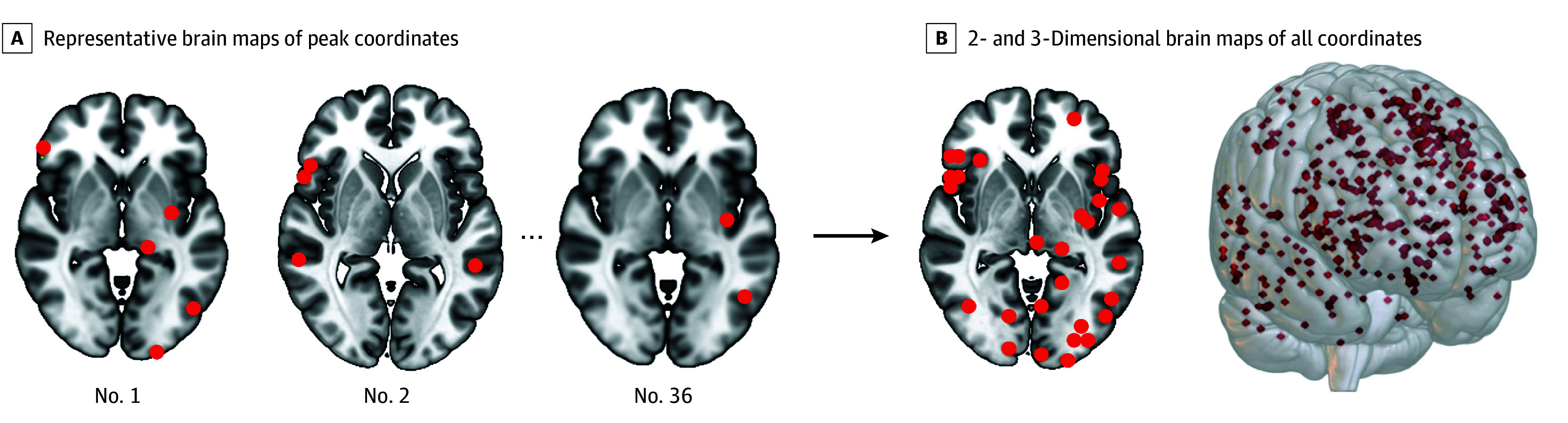
Neuroimaging Studies on Creativity Displaying High Heterogeneity of Results A, Brain maps showing peak coordinates of task activation (red) from individual studies of creativity (showing results from 3 of 36 studies). B, Brain maps showing all included coordinates.

**Figure 2.**
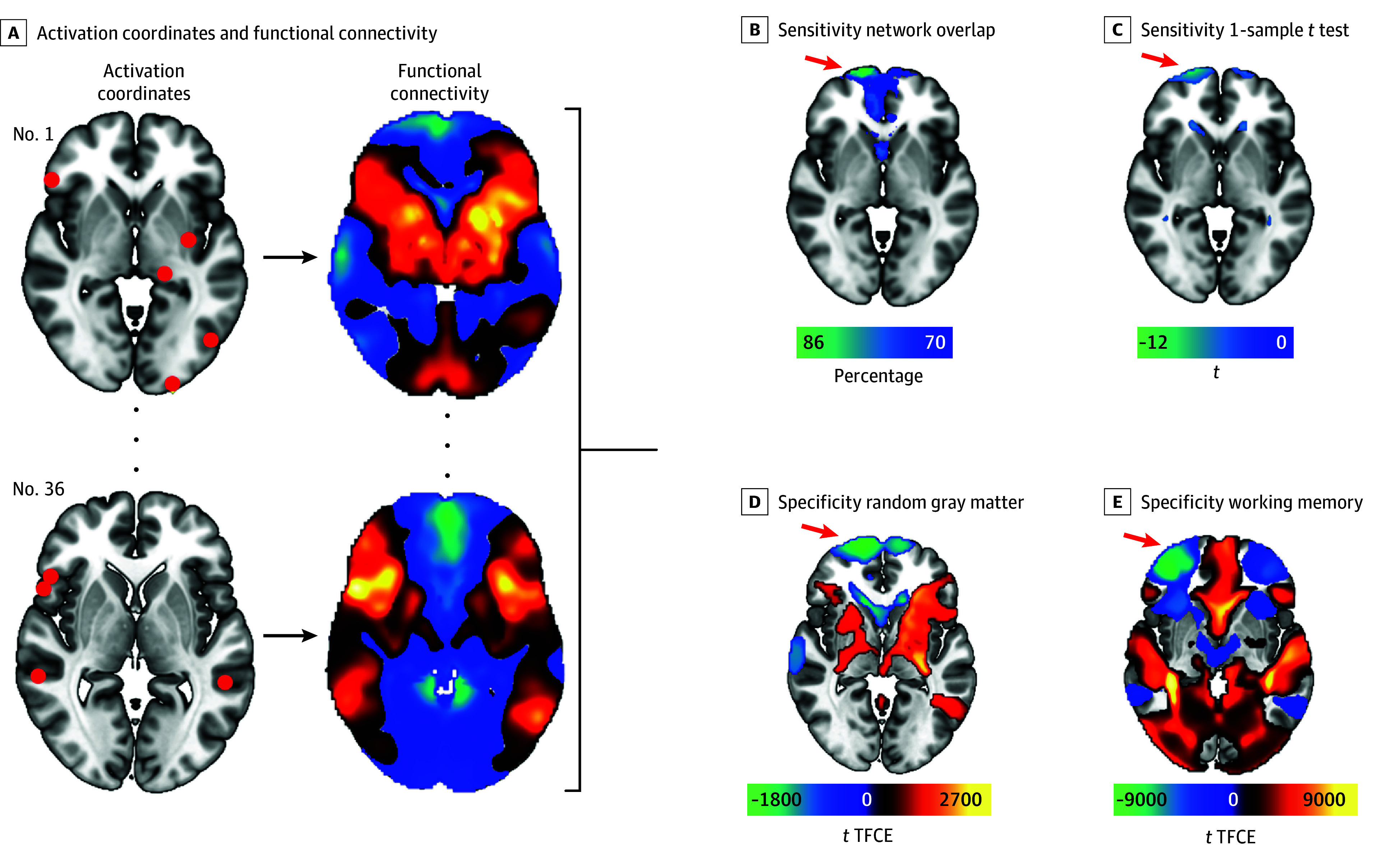
Methodological Overview A, Brain maps showing peak coordinates of task activation (red) from individual studies of creativity were used as seed regions. For each study/seed, functional connectivity maps were obtained using a 1000-participants resting-state functional connectome. B, Individual connectivity maps were thresholded (*t* ≥ 5) and binarized to obtain a network overlap map in which peak overlaps indicate the regions most consistently connected to the activation seeds. C, Significant results from a 1-sample *t* test at a family-wise error rate–corrected *P* value threshold of <.05. D and E, Specificity was assessed via statistical comparison with permutation analysis of linear models at 10 000 permutations against random gray matter and working memory coordinates. Reported clusters are significant at false discovery rate–corrected *P* < .05 with threshold-free cluster enhancement (TFCE). The right frontal pole (red arrows) was a consistent finding across all sensitivity and specificity analyses.

In total, 415 brain coordinates activated by creativity tasks (vs control tasks) were identified, taken from 36 individual studies involving 857 participants (pooled mean [SD] age, 24.1 [6.91] years; 461 [54%] female and 396 [46%] male). Demographic information on these participants by study can be found in eTable 1 in Supplement 1. These coordinates were highly heterogenous across different studies (Figure 1). However, 86% of these studies reported coordinates that were part of a common brain circuit (Figure 2). This circuit was defined by negative functional connectivity to the right frontal pole (Montreal Neurological Institute coordinates: *x* = 20, *y* = 68, and *z* = –6). This circuit finding was robust to methodological variation, including varying the sphere size at each coordinate (eFigure 1 in Supplement 1), running the analysis on individual-level or group-level coordinates rather than study-level coordinates (eFigure 2 in Supplement 1), varying the statistical cutoffs (eFigure 1, eTable 2 in Supplement 1), or analyzing divergent thinking and motoric creativity separately (eFigure 7 in Supplement 1). This result was also specific to creativity coordinates compared with random coordinates or to working memory coordinates (false discovery rate, *P* < .05) (Figure 2D-E). Results were again significant independent of whether we studied individual-level, study-level, or group-level coordinates (eFigure 2 in Supplement 1).

**Figure 3.**
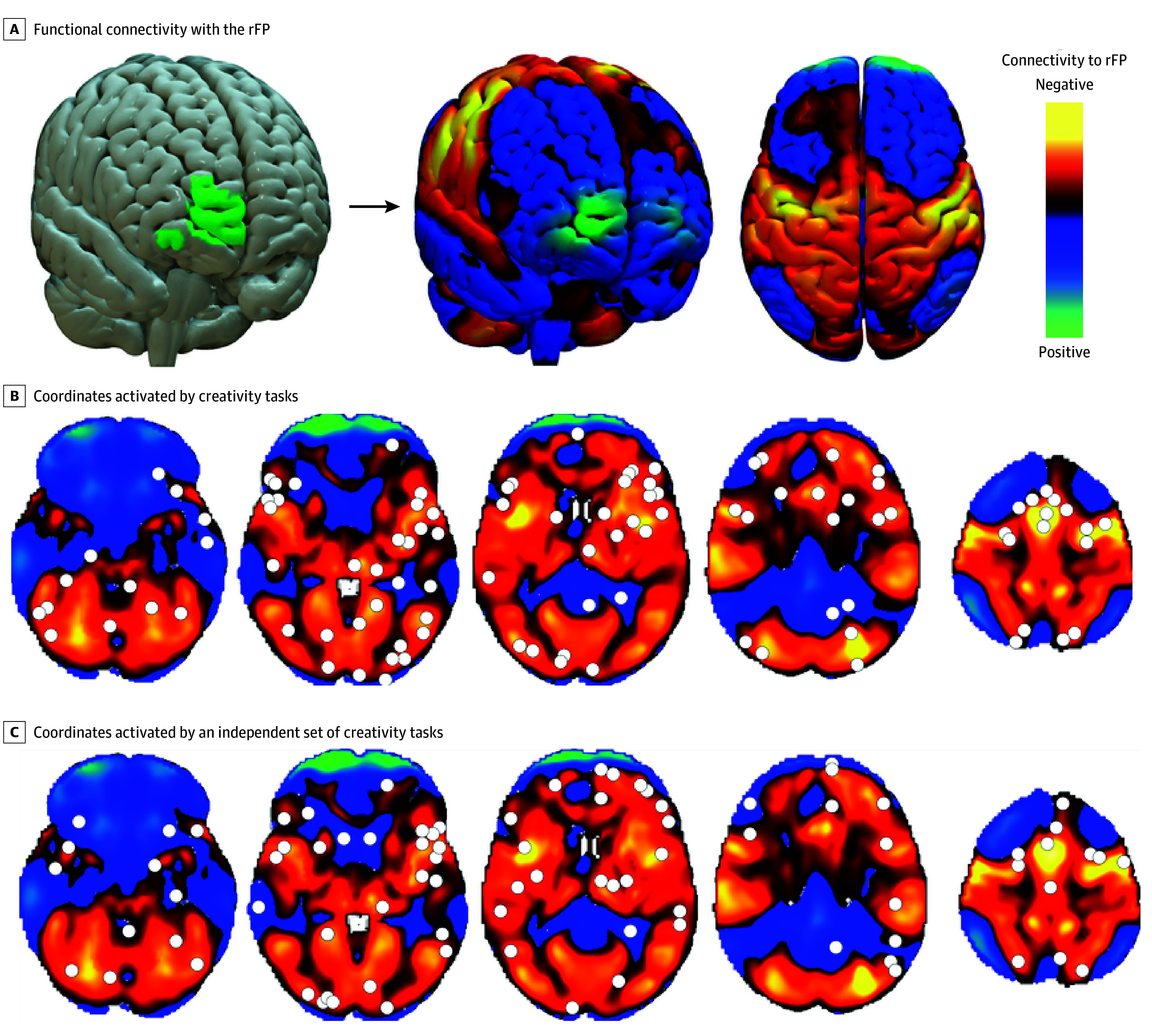
Connectivity of Creativity Activation Foci to the Right Frontal Pole (rFP) A, Functional connectivity with the rFP (green, left) defines a distributed brain circuit (right) that will encompass coordinates activated by creativity tasks. B, Brain maps showing coordinates activated in 36 studies of creativity (white dots) fall within the circuit (warm colors) defined by negative functional connectivity to the rFP. C, Brain maps showing coordinates activated in 30 independent studies of creativity (white dots) fall within the same circuit.

Based on these results, functional connectivity with the right frontal pole (Figure 3A) defined a brain circuit that would best encompass coordinates activated by creativity tasks (Figure 3B), but not random coordinates or coordinates activated by working memory tasks. We herein refer to this map of connectivity with the rFP as the creativity circuit. Activation coordinates from 30 independent studies (eTable 3 in Supplement 1) of creative tasks also overlapped with this circuit (Figure 3C), an overlap that was significantly stronger than expected by chance (*t*_548_ = –2.75; *P* = .007). Repeating our data-driven network mapping analysis using this independent dataset also showed peak overlap of negative connectivity in the right frontal pole (eFigure 3 in Supplement 1).

**Figure 4.**
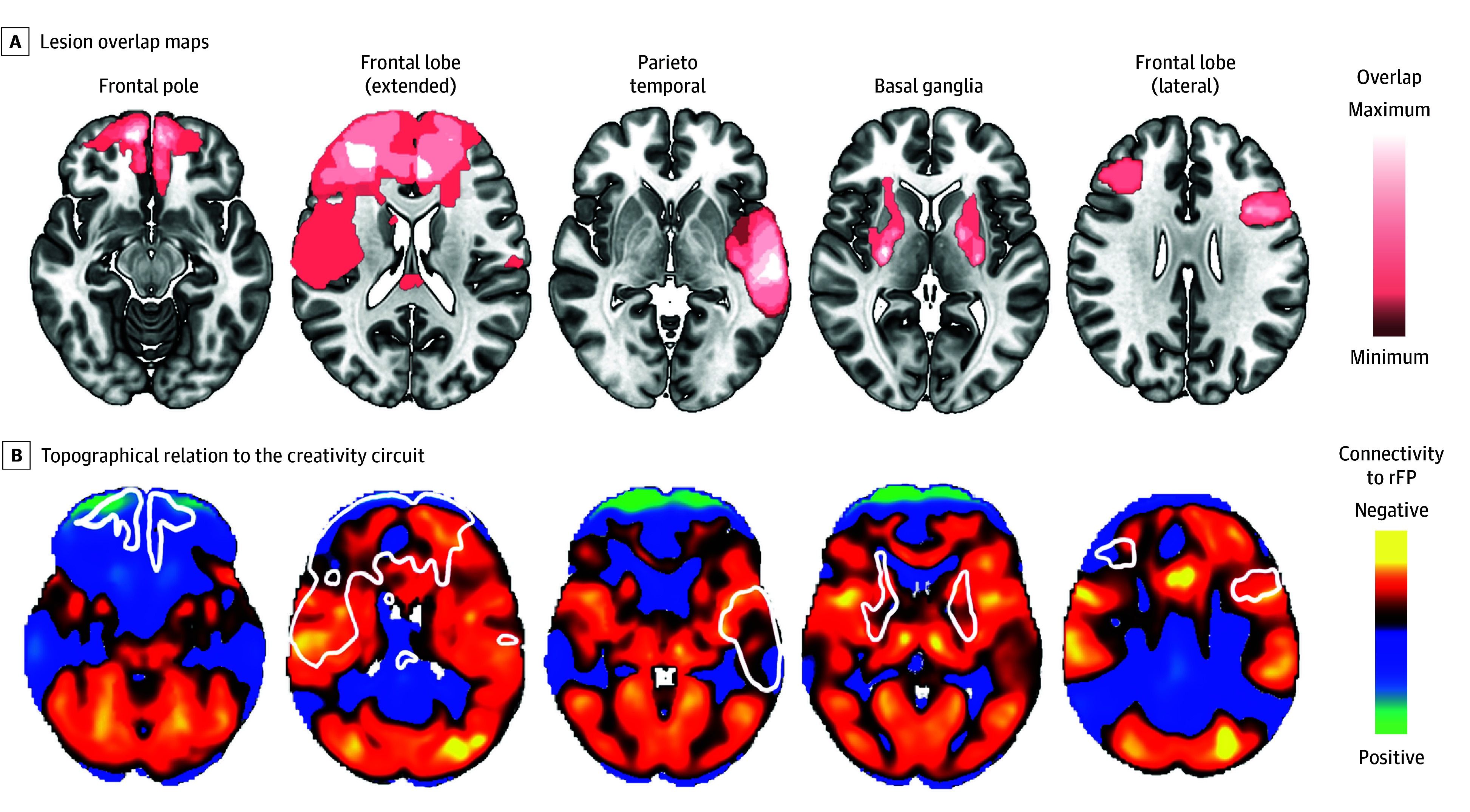
Lesions Affecting Creativity Task Performance and the Creativity Circuit A, Lesion overlap maps from Abraham et al^31^ showing groups of patients with lesions in different neuroanatomical locations. Patients with lesions to the lateral frontal lobe (right) had impaired performance on creativity tasks, while patients with lesions to the frontal pole (left) had higher performance. B, Topographical relation of overlap maps in (A, white outlines) to the creativity circuit. Intersection of the lesion locations with our creativity circuit was significantly associated with lesion-induced effects on creativity task performance (eFigure 4 in Supplement 1). rFP indicates right frontal pole.

We next tested whether damage to the identified creativity circuit from brain disease aligned with reported effects on creative abilities. First, we analyzed data from 5 groups of patients with brain lesions who previously underwent a battery of creativity tasks^31^ (Figure 4A). These 5 lesion groups differed in their creative abilities, with the group having a lateral frontal lobe lesion showing the largest impairment in creativity and the group with a frontal pole lesion showing the least impairment in creativity and even an increase in creativity, although that result was not statistically significant.^31^ Consistent with these findings, we found that these lesion groups intersected our creativity circuit to varying degrees (*F*_4,175_ = 10.25; *P* < .001) (Figure 4B). The group with a lateral frontal lesion intersected regions negatively connected to the rFP, similar to regions activated by creativity tasks and consistent with their lesion-induced deficits in creativity. By contrast, the group with a frontopolar lesion intersected regions positively connected to the rFP and consistent with their lack of impairment and potential increase in creativity. Post hoc *t* tests confirmed a significant difference in the degree to which these 2 lesion groups intersected our creativity network (*t*_70_ = –0.32; *P* < .001) (eFigure 4B in Supplement 1).

**Figure 5.**
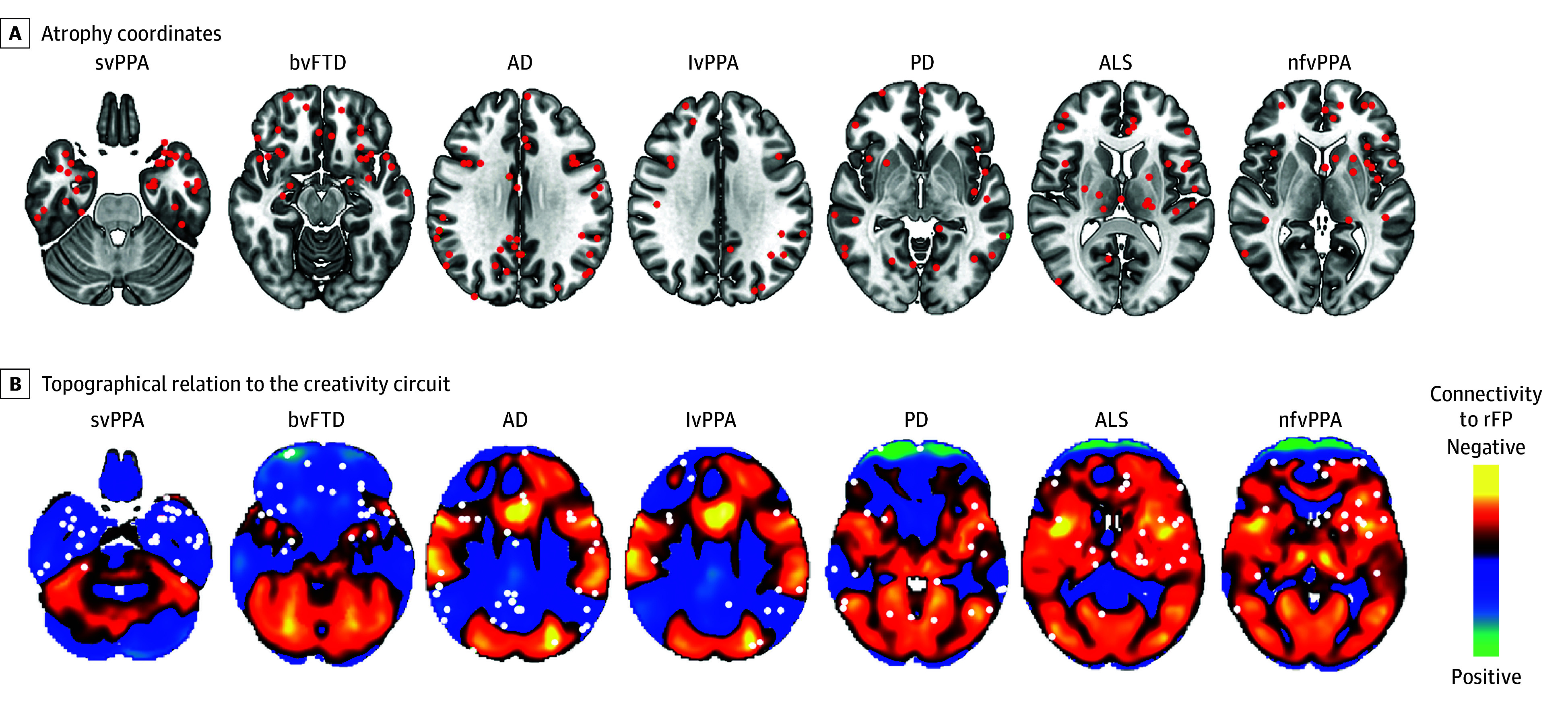
Neurodegenerative Atrophy Patterns and the Creativity Circuit A, Brain maps showing atrophy coordinates (red) in different neuroanatomical locations in studies on semantic (svPPA), logopenic (lvPPA), and nonfluent (nfvPPA) variants of primary progressive aphasia, behavioral variant of frontotemporal dementia (bvFTD), typical Alzheimer disease (AD), Parkinson disease (PD), and amyotrophic lateral sclerosis (ALS). Patients with svPPA and bvFTD (left) have been reported to experience increases in creativity, while patients with nfvPPA (right) have been reported to experience decreased creativity. B, Topographical relation of atrophy coordinates in panel A to the creativity circuit (eFigure 5B in Supplement 1). For svPPA, 75% of the atrophy coordinates hit regions positively connected to the right frontal pole (rFP) (cool colors). By contrast, for nfvPPA, 68% of the atrophy coordinates hit regions negatively connected to the rFP (warm colors).

Next, we analyzed the location of brain atrophy across 7 different neurodegenerative disorders, including 2 disorders previously associated with increased creativity (svPPA and behavioral variant of frontotemporal dementia [bvFTD]) (Figure 5). Coordinates of brain atrophy from these disorders differed in the degree to which they aligned with the creativity circuit (*F*_6,1295_ = 6.725; *P* < .001) (eFigure 5B in Supplement 1). Post hoc 2-sample *t* tests showed that this difference was associated with stronger alignment with svPPA than with other conditions, including nonfluent/agrammatic variant primary progressive aphasia (nfvPPA; *t*_30_ = 0.18; *P* < .001), Parkinson disease (*t*_70_ = 0.08; *P* = .04), and amyotrophic lateral sclerosis (*t*_48_ = 0.11; *P* = .003). There was also stronger alignment with bvFTD compared with nfvPPA (*t*_34_ = 0.15; *P* < .001) (eFigure 5B in Supplement 1).

Finally, we examined the intersection between our creativity circuit and the location of brain atrophy in patients with frontotemporal dementia (FTD) and new-onset creativity.^12^ We found that the location of brain atrophy in these patients was negatively functionally connected to the creativity-related activation foci (*t*_35_ = –2.14; Cohen *d* = –0.36; *P* = .04). By contrast, the mean connectivity between noncreative FTD atrophy patterns and creativity activation foci was not significantly different from 0 (*t*_35_ = 0.17; Cohen *d* = 0.03; *P* = .86) (eFigure 6 in Supplement 1).

## Discussion

Using network mapping of meta-analytic data, this study found that coordinates activated by creativity tasks map to a common brain circuit, defined by negative connectivity to the right frontal pole. The topography of this circuit aligned with lesion-induced effects on creativity and locations of brain atrophy associated with creativity changes in neurodegenerative diseases.

Previous neuroimaging studies of healthy participants suggest that the brain regions activated by creative tasks differ depending on the specific task or creative domain.^21^ Here, we show that these heterogenous locations of brain activation map to a common brain circuit. Our findings do not preclude interesting differences across tasks or creative domains but rather suggest that despite regional differences there is a common neural substrate for creativity when assessed at the brain circuit level.

This brain circuit for creativity was defined by negative functional connectivity between coordinates activated by creativity tasks and the right frontal pole. Although the interpretation of negative functional connections remains debated,^53^ negative connections are often observed between brain regions activated by tasks and other brain regions deactivated by tasks. Thus, it is possible that creativity tasks activate different brain regions but deactivate a common region in the right frontal pole. Consistent with this hypothesis, past neuroimaging research has found lower activity in prefrontal regions when individuals were asked to improvise or generate ideas compared with evaluation of ideas.^28,54,55^ Other studies have implicated medial prefrontal regions near the frontopolar cortex in creative thinking,^9,56^ including a study showing negative signal change when planning a creative act.^56^ Because most task-based fMRI studies only report coordinates of activation, not coordinates of deactivation, it is possible that deactivation of the frontopolar cortex during creativity tasks is more common than currently appreciated.^20^

Another possibility is that the right frontal pole is a hub that defines the circuit of brain regions likely to be activated by creativity tasks, but it is not itself deactivated by creativity tasks. Either way, neuroimaging studies of task modulation cannot determine whether the right frontal pole plays a causal role in creativity. For causal inference, one must turn to other complementary data sources, such as patients with brain lesions.

Our coordinate-based brain circuit set a testable hypothesis for lesion studies: If creativity involves deactivating the right frontal pole, lesions to this region (compared with other regions) should increase creativity. Although there have been very few lesion-based studies of creativity,^57^ our analysis of available lesion data supports this hypothesis. Participants with frontopolar lesions performed better than patients with lesions in other brain locations and better than healthy controls in 1 of the creativity tasks that was measured.^31^

The topography of our circuit also aligned with the changes of creativity reported among some patients with neurodegenerative diseases.^12^ Specifically, our circuit aligned with increased creativity reported in svPPA and bvFTD and also aligns with a single case report of decreases in creativity in nfvPPA.^58^ In their recent study, Friedberg et al^12^ identified a dorsomedial occipital region to be anticorrelated with atrophy in patients with new creativity onset. While this region was not specific to creativity in our analysis, it aligned with the coordinate-based creativity circuit we found. Our result expands this finding to the effect that the visual cortex is part of the network of brain regions activated by creative tasks, but not the most sensitive and specific region involved in creative behavior across domains. Furthermore, in line with the findings of Friedberg et al,^12^ atrophy patterns in svPPA most strongly aligned with the creativity circuit.

Although consistent across task activation, lesions, and neurodegenerative disease, it remains unclear why the hub of our circuit is in the right frontal pole or what role this region may play in mediating creativity. Although speculative, our results suggest that the right frontal pole may work to actively suppress creativity. Deactivation of this region during creativity tasks, damage to this region by brain lesions, or damage to this region in neurodegenerative disease may lead to a release of creativity, a process termed *paradoxical functional facilitation*.^59^ Creativity is thought to involve different steps, including initial free association or idea generation followed by idea selection and refinement, the latter of which may involve analytic or self-censoring assessments.^60,61^ Our circuit results may identify a neuroanatomical substrate for this latter, evaluative step. Specifically, deactivation of the frontal pole may result in decreased self-monitoring and a release of disinhibited creative output^62^ or spontaneous improvisation.^54^ Alternatively, the right frontal pole has been implicated in high-level cognitive control and constraining novelty seeking.^63^ Transcranial magnetic stimulation inhibition of the right frontopolar cortex can result in increased novelty seeking.^64^ Effects of transcranial magnetic stimulation to the right frontal pole on creativity have yet to be conducted, but this is a testable hypothesis for future research.

Our study is not the first attempt at mapping creativity to a brain circuit, and our results complement previous circuit approaches to mapping creativity.^25,26,27,28,29^ This prior work identified correlations between brain network connectivity and creativity and in 1 case^29^ validated their findings with brain stimulation. By contrast, our study identified a brain circuit that encompasses coordinates activated by creativity tasks and validated the findings using lesion and atrophy locations. Future work is needed to reconcile these different methods and results and to relate these neuroanatomical findings to an extensive literature on theoretical models of creativity.^10,61,65^

### Limitations

There are several important limitations to our work. First, our maps are based on fMRI activation data that draw on a binarized creativity measure in which tasks were considered either creative or noncreative. This may oversimplify the complex structure of creativity unfolding over time or nuances of individual studies. Second, the data used to derive the creativity circuit are limited to existing literature on fMRI creativity tasks. Not all creative behavior can be studied in an fMRI scanner. Hence, there may be cases in which creativity is not negatively linked to the right frontal pole. Third, our study focused on identifying a common substrate across creativity domains, but this does not preclude important differences specific to different forms of creativity.^22,23,35^ There are many different creativity domains, creativity is likely different in different individuals, and inhibiting the frontal pole is unlikely to account for the unique and diverse forms of creativity. Fourth, our approach relies on retrospective neuroimaging data. Prospective validation of the creativity circuit, especially using causal sources of evidence, such as brain lesions or brain stimulation, is needed.^66^ Fifth, using working memory as a control condition allows us to increase specificity to core components of creativity, such as originality or novelty, but may mask other important contributors to creativity, such as working memory and intelligence.^24,67,68^ Finally, since our method analyzes data from task-based fMRI activation studies that use different imaging processing methods, statistical methods, and study sample characteristics, potential noise associated with this heterogeneity may impact our results. However, this heterogeneity should bias against finding a convergent brain circuit for creativity.

### Conclusions

The results of this study using network mapping of meta-analytic data suggest that heterogenous coordinates activated by creativity tasks map onto a common brain circuit. This circuit aligns with the effects of brain lesions and neurodegenerative diseases on creativity and provides testable hypotheses for future research.

## Notes

Supplement 1.
eMethods
**eTable 1.** Studies reporting fMRI activation coordinates for creative tasks (> control tasks), as initially found and analyzed by Brown & Kim (2021) **eFigure 1.** The topography of the network overlap map is robust across different t-thresholds and sphere sizes **eTable 2.** Spatial Pearson correlations between network overlap maps for the t-thresholds 5, 7, and 9 and across different sphere sizes (0 mm, 4 mm, 8 mm) **eFigure 2.** Coordinate Network Mapping (CNM) of creativity-specific activation foci on three different levels **eTable 3.** Additional studies reporting fMRI activation coordinates for creative tasks (not controlled for task-based activation) as initially found and analyzed by Chen et al. (2020) and Gonen-Yaacovi et al. (2013) **eTable 4.** Studies reporting fMRI activation coordinates for working memory as initially found by Rottschy et al. (2012) and Wang et al. (2019) **eFigure 3.** Replicating the coordinate network mapping approach for these independent sets of coordinates, we find a significant sensitive and specific spot close to and partly overlapping with the right frontopolar hub region of the creativity circuit (outlined in red in the conjunction map) **eTable 5.** Studies reporting atrophy coordinates for several neurodegenerative diseases including behavioral variant of frontotemporal dementia (bvFTD), semantic (svPPA), logopenic (lvPPA) and non-fluent (nfvPPA) variants of primary progressive aphasia, Parkinson’s disease (PD), Alzheimer’s disease (AD), and amyotropic lateral sclerosis (ALS) **eFigure 4.** Lesions affecting creativity task performance and the creativity circuit **eFigure 5.** Neurodegenerative atrophy patterns and the creativity circuit **eFigure 6.** Scatter plot displaying the bell curve for average ROI-to-ROI correlations between 36 creativity seeds and traced atrophy for FTD patients displaying emergence of visual artistic creativity (orange) and FTD patients not displaying emergence of visual artistic creativity (blue) **eFigure 7.** Network overlap computed for motoric creativity and divergent thinking separately **eFigure 8.** PRISMA diagram replicating ALE meta-analysis conducted by Brown & Kim (2021)

Supplement 2.
Data Sharing Statement
