# Supplementary material for: Mapping Neuroimaging Findings of Creativity and Brain Disease Onto a Common Brain Circuit
Source: JAMA Netw Open. 2025 Feb 13;8(2):e2459297. doi: 10.1001/jamanetworkopen.2024.59297 (PMC11826368; doi:10.1001/jamanetworkopen.2024.59297)
Supplement: Supplement 1. — eMethods eTable 1. Studies reporting fMRI activation coordinates for creative tasks (> control tasks), as initially found and analyzed by Brown & Kim (2021) eFigure 1. The topography of the network overlap map is robust across different t-thresholds and sphere sizes eTable 2. Spatial Pearson correlations between network overlap maps for the t-thresholds 5, 7, and 9 and across different sphere sizes (0 mm, 4 mm, 8 mm) eFigure 2. Coordinate Network Mapping (CNM) of creativity-specific activation foci on three different levels eTable 3. Additional studies reporting fMRI activation coordinates for creative tasks (not controlled for task-based activation) as initially found and analyzed by Chen et al. (2020) and Gonen-Yaacovi et al. (2013) eTable 4. Studies reporting fMRI activation coordinates for working memory as initially found by Rottschy et al. (2012) and Wang et al. (2019) eFigure 3. Replicating the coordinate network mapping approach for these independent sets of coordinates, we find a significant sensitive and specific spot close to and partly overlapping with the right frontopolar hub region of the creativity circuit (outlined in red in the conjunction map) eTable 5. Studies reporting atrophy coordinates for several neurodegenerative diseases including behavioral variant of frontotemporal dementia (bvFTD), semantic (svPPA), logopenic (lvPPA) and non-fluent (nfvPPA) variants of primary progressive aphasia, Parkinson’s disease (PD), Alzheimer’s disease (AD), and amyotropic lateral sclerosis (ALS) eFigure 4. Lesions affecting creativity task performance and the creativity circuit eFigure 5. Neurodegenerative atrophy patterns and the creativity circuit eFigure 6. Scatter plot displaying the bell curve for average ROI-to-ROI correlations between 36 creativity seeds and traced atrophy for FTD patients displaying emergence of visual artistic creativity (orange) and FTD patients not displaying emergence of visual artistic creativity (blue) eFigure 7. Network overlap co [file jamanetwopen-e2459297-s001.pdf]

Supplementary Online Content

Kutsche J, Taylor JJ, Erkkinen MG, et al. Mapping neuroimaging findings of creativity and brain disease onto a common brain circuit. *JAMA Netw Open*. 2025;8(2):e2459297. doi:10.1001/jamanetworkopen.2024.59297

eMethods
eTable 1. Studies reporting fMRI activation coordinates for creative tasks (> control tasks), as initially found and analyzed by Brown & Kim (2021)
eFigure 1. The topography of the network overlap map is robust across different t-thresholds and sphere sizes
eTable 2. Spatial Pearson correlations between network overlap maps for the t-thresholds 5, 7, and 9 and across different sphere sizes (0 mm, 4 mm, 8 mm)
eFigure 2. Coordinate Network Mapping (CNM) of creativity-specific activation foci on three different levels
eTable 3. Additional studies reporting fMRI activation coordinates for creative tasks (not controlled for task-based activation) as initially found and analyzed by Chen et al. (2020) and Gonen-Yaacovi et al. (2013)
eTable 4. Studies reporting fMRI activation coordinates for working memory as initially found by Rottschy et al. (2012) and Wang et al. (2019)
eFigure 3. Replicating the coordinate network mapping approach for these independent sets of coordinates, we find a significant sensitive and specific spot close to and partly overlapping with the right frontopolar hub region of the creativity circuit (outlined in red in the conjunction map)
eTable 5. Studies reporting atrophy coordinates for several neurodegenerative diseases including behavioral variant of frontotemporal dementia (bvFTD), semantic (svPPA), logopenic (lvPPA) and non-fluent (nfvPPA) variants of primary progressive aphasia, Parkinson's disease (PD), Alzheimer's disease (AD), and amyotropic lateral sclerosis (ALS)
eFigure 4. Lesions affecting creativity task performance and the creativity circuit
eFigure 5. Neurodegenerative atrophy patterns and the creativity circuit
eFigure 6. Scatter plot displaying the bell curve for average ROI-to-ROI correlations between 36 creativity seeds and traced atrophy for FTD patients displaying emergence of visual artistic creativity (orange) and FTD patients not displaying emergence of visual artistic creativity (blue)
eFigure 7. Network overlap computed for motoric creativity and divergent thinking separately
eFigure 8. PRISMA diagram replicating ALE meta-analysis conducted by Brown & Kim (2021)

This supplementary material has been provided by the authors to give readers additional information about their work.

© 2025 Kutsche J et al. *JAMA Network Open*.

eMethods

Study data

We extracted 415 activation coordinates from 36 fMRI studies on creativity that were used in a recent ALE meta-analysis conducted based on PRISMA guidelines (for a replication of methods see eFigure 8).[1] We selected this meta-analysis because it was the most recent and comprehensive one, included control conditions for each creative task, and included diverse forms of artistic creativity, including music improvisation, drawing, writing, or dancing, as well as divergent thinking. Any coordinates reported in Talairach space were transformed into MNI space using the Lancaster transform function in GingerALE version 3.0.2.[2,3,4] For every study, we created 4 mm diameter spheres[5,6] at each coordinate activated by *creative vs non-creative tasks*. For our primary analysis, coordinates from each study were combined into single study-level maps[5] (Figure 1). However, we repeated our analysis considering each coordinate independently and considering only group-level coordinates that were significant in ALE meta-analysis. All included study data is previously published, publicly available (eTables 1, 3, 4, 5) with patient consent per individual journal requirements. The study was approved by Mass General Brigham/Partners Institutional Review Board Protocol 2020P002987.

Coordinate Network Mapping

In line with previous work[5,6,7], we used a method termed *coordinate network mapping* to identify the network of brain regions functionally connected to each of the 36 study-level seeds, using a resting-state functional connectome from a large cohort of healthy subjects (n = 1,000). Each of the individual 36 obtained functional connectivity maps was thresholded ($t \geq 5$)[8], binarized and overlayed to obtain a *network overlap map* that indicates areas of most consistent connectivity across all individual maps. Following the approach of Peng et al. (2022)[5] and Taylor et al. (2023)[7], we also ran a 1-sample *t*-test on these maps to identify connections that were statistically consistent (FWE-corrected $p < 0.05$). To ensure results were robust to methodological variation, we repeated this procedure for single-voxel coordinates and 8 mm spheres as well as *t*-thresholds of 7 and 9 (see eFigure 1).

Specificity

Although reported activation coordinates were already controlled for non-creative tasks in the original studies, it remains possible that some coordinates are more likely to appear in neuroimaging contrasts than other coordinates, and that some brain regions are more likely to be functionally connected to any given set of coordinates. To control for these possibilities, we employed two specificity analyses, similar to prior coordinate network mapping studies[5,6], using (1) 415 random grey matter coordinates and (2) 3,072 coordinates activated by working memory tasks. We selected working memory as a control condition because it requires attention, cognitive effort and manipulation, thus controlling for executive functions that are an important component of many creativity tasks[9,10] but are not generally considered core components of creativity such as originality or novelty.[11,12,13] For the random grey matter coordinates, we combined 415 random coordinates so that for each of the 36 combined study-level seeds for creativity there was a pairwise matched combined seed of random coordinates. Similarly, 150 combined seeds for working memory coordinates were obtained, drawing on two prior ALE meta-analyses (see eTable 4).[14,15] Connections specific to creativity seeds versus these control seeds (FDR-corrected $p < 0.05$) were obtained through a 2-sample *t*-test using *Permutation Analysis of Linear Models* (PALM)[16] at 10,000 permutations and threshold-free cluster enhancement (TFCE[17]).

To identify connections sensitive and specific to creativity across all analyses, we performed a conjunction analysis of four maps: (1) the network overlap map (thresholded at 86 %), (2) significant voxels from the 1-sample *t*-test (FWE-corrected $p < 0.05$), (3) significant voxels from the two-sample *t*-test versus random coordinates (FDR-corrected $p < 0.05$), and (4) significant voxels from the 2-sample *t*-test against working memory coordinates (FDR-corrected $p < 0.05$). The set of voxels that satisfied the above four criteria was considered the "hub region" of our creativity circuit.

We repeated the sensitivity and specificity analyses on the ALE coordinate-level, using the reported peak coordinates of the initial ALE meta-analysis, as well as on the individual coordinate level and repeated the conjunction analysis to demonstrate generalizability of our results across levels of analysis (see eFigure 2).

To test for reproducibility of our creativity circuit across independent datasets, we extracted 383 coordinates activated by creativity tasks from 30 independent studies found through two other ALE meta-analyses[12,18] in which studies without non-creative control tasks were included as well. We then assessed how many of those coordinates are negatively connected to the hub region of the creativity circuit. We further repeated the CNM method described above for the independent coordinates to assess similarities in connectivity outcome. Additionally, a 2-sample *t*-test between the group of *t*-values of the creativity circuit circumscribed by the independent creativity coordinates and the group of *t*-values circumscribed by a set of 383 random grey matter

© 2025 Kutsche J et al. *JAMA Network Open*.

coordinates was run to show that the *t*-value distribution is significantly different from a random distribution ($p < 0.05$).

Alignment with lesion effects

To test for alignment between our creativity circuit and lesion effects on creativity, we obtained lesion overlap maps (n = 56 patients) from a previous study that examined the relationship between lesion location and performance on creativity tasks.[19] Although lesions locations from each individual patient were not available, lesion overlap maps for five sets of patients grouped by lesion location were available: lateral prefrontal, frontal polar, frontal lobe (large lesions extending beyond specific prefrontal regions), basal ganglia, and parietotemporal. First, we qualitatively examined the intersection of each lesion group with our creativity circuit. Second, we quantitatively calculated functional connectivity between each of the 5 lesion overlap maps to each of the 36 creativity study-level seeds. Resulting *r*-values were converted to a normal distribution using the Fisher-*z*-transformation. A two-tailed 1-sample *t*-test on the resulting values indicated distributions significantly different from 0 ($p < 0.05$). An ANOVA and post-hoc 2-sample *t*-tests showed significant group-level differences (Bonferroni-corrected $p < 0.001$).

Alignment with neurodegenerative disease

Atrophy coordinates from groups of patients: To test for alignment between our creativity circuit and locations of brain atrophy in patients with neurodegenerative disorders, we extracted atrophy coordinates from neuroimaging studies of patients with 7 different diagnoses: behavioral variant of frontotemporal dementia (bvFTD, 24 studies, 348 coordinates, n=462), semantic variant of primary progressive aphasia (svPPA, 19 studies, 178 coordinates, n=292), non-fluent variant of PPA (nfvPPA, 13 studies, 189 coordinates, n=173) logopenic variant of PPA (lvPPA, 12 studies, 188 coordinates, n=175), typical Alzheimer's disease (AD, 37 studies, 568 coordinates, n=825), amyotrophic lateral sclerosis (ALS, 31 studies, 350 coordinates, n=773), and Parkinson's disease (PD, 53 studies, 441 coordinates, n=2,104) (for a full list of all studies that were included, see eTable 5). To avoid bias, the list of studies for each diagnosis was taken from recent ALE meta-analyses.[20,21,22,23,24,25] Since svPPA and bvFTD are most commonly reported to lead to creativity increases[26,27], we hypothesized that the atrophy profiles of these diseases would better align with the creativity network than atrophy from other neurodegenerative conditions. First, we qualitatively examined the intersection of the atrophy coordinates with our creativity circuit. Second, we quantitatively computed the functional connectivity between atrophy study-seeds and the hub of the creativity circuit in the right frontal pole. Two-tailed 1-sample *t*-tests on each group indicated distributions significantly different from 0 ($p < 0.05$). An ANOVA and post-hoc 2-sample *t*-tests showed significant group-level differences (Bonferroni-corrected $p < 0.001$).

Atrophy locations associated with new creativity onset: To complement the above analysis focused on general group-level atrophy coordinates, we traced the location of brain atrophy from patients with frontotemporal dementia and increased artistic creativity.[27] Each traced atrophy location was evaluated for accuracy by two experienced neurologists (MGE and IK). Connectivity between each lesion location and our 36 study-level maps of coordinates activated by creativity tasks was computed, identical to our analysis of lesion locations. Two-tailed 1-sample *t*-tests were run to indicate whether average connectivity values were significantly different from 0 ($p < 0.05$).

References

[1] Brown S, Kim E. The Neural Basis of Creative Production: A Cross-Modal Ale Meta-Analysis. *Open Psychology.* 2021;3:103–32. doi:10.1515/psych-2020-0114.
[2] Eickhoff SB, Laird AR, Grefkes C, Wang LE, Zilles K, Fox PT. Coordinate-based activation likelihood estimation meta-analysis of neuroimaging data: A random-effects approach based on empirical estimates of spatial uncertainty. *Human Brain Mapping.* 2009;30:2907–2926. doi:10.1002/hbm.20718.
[3] Eickhoff SB, Bzdok D, Laird AR, Kurth F, Fox PT. Activation likelihood estimation revisited. *Neuroimage.* 2012;59:2349–2361. doi:10.1016/j.neuroimage.2011.09.017.
[4] Turkeltaub PE, Eickhoff SB, Laird AR, Fox M, Wiener M, Fox P. Minimizing within-experiment and within-group effects in activation likelihood estimation meta-analyses. *Human Brain Mapping.* 2012;33:1–13. doi:10.1002/hbm.21186.
[5] Peng S, Xu P, Jiang Y, Gong G. Activation network mapping for integration of heterogeneous fMRI findings. *Nature Human Behaviour.* 2022;6:1417–1429. doi:10.1038/s41562-022-01371-1.
[6] Stubbs JL, Taylor JJ, Siddiqi SH, et al. Heterogeneous neuroimaging findings across substance use disorders localize to a common brain network. *Nature Mental Health.* 2023;1:772–781. doi:10.1038/s44220-023-00128-7.

© 2025 Kutsche J et al. *JAMA Network Open.*

[7] Taylor JJ, Lin C, Talmasov D, et al. A transdiagnostic network for psychiatric illness derived from atrophy and lesions. *Nature Human Behaviour*. 2023;7: 420429. doi:10.1038/s41562-022-01501-9.
[8] Cohen AL, Fox MD. Reply: The Influence of Sample Size and Arbitrary Statistical Thresholds in Lesion-Network Mapping. *Brain*. 2020;143(5):e41. doi:10.1093/brain/awaa095.
[9] Miroshnik KG, Forthmann B, Karwowski M, Benedek M. The relationship of divergent thinking with broad retrieval ability and processing speed: A meta-analysis. *Intelligence*. 2023;98101739. doi:10.1016/j.intell.2023.101739.
[10] Benedek M, Jauk E, Sommer M, Arendasy M, Neubauer AC. Intelligence, creativity, and cognitive control: The common and differential involvement of executive functions in intelligence and creativity. *Intelligence*. 2014;46:73–83. doi:10.1016/j.intell.2014.05.007.
[11] Dow GT. Defining creativity. In: Plucker J, ed. *Creativity and Innovation: Theory, Research, and Practice*. Prufrock Press. 2016:5–22. doi:10.4324/9781003233930-2.
[12] Gonen-Yaacovi G, de Souza LC, Levy R, Urbanski M, Josse G, Volle E. Rostral and caudal prefrontal contribution to creativity: a meta-analysis of functional imaging data. *Frontiers in Human Neuroscience*. 2013;7465. doi:10.3389/fnhum.2013.00465.
[13] Acar S, Burnett C, Cabra JF. Ingredients of Creativity: Originality and More. *Creativity Research Journal*. 2017;29(2):133–144. doi:10.1080/10400419.2017.1302776.
[14] Rottschy C, Langner R, Dogan I, et al. Modelling neural correlates of working memory: A coordinate-based meta-analysis. *NeuroImage*. 2012;60:830–846. doi:10.1016/j.neuroimage.2011.11.050.
[15] Wang H, He W, Wu J, Zhang J, Jin Z, Li L. A coordinate-based meta-analysis of the n-back working memory paradigm using activation likelihood estimation. *Brain and Cognition*. 2019;132:1–12. doi:10.1016/j.bandc.2019.01.002.
[16] Winkler AM, Ridgway GR, Webster MA, Smith SM, Nichols TE. Permutation inference for the general linear model. *Neuroimage*. 2014;92(100):381–97. doi:10.1016/j.neuroimage.2014.01.060.
[17] Smith SM, Nichols TE. Threshold-free cluster enhancement: addressing problems of smoothing, threshold dependence and localisation in cluster inference. *Neuroimage*. 2009;44(1):83-98. doi:10.1016/j.neuroimage.2008.03.061.
[18] Chen Q, Beaty RE, Qiu J. Mapping the artistic brain: Common and distinct neural activations associated with musical, drawing, and literary creativity. *Human Brain Mapping*. 2020;41:3403–3419. doi:10.1002/hbm.25025.
[19] Abraham A, Beudt S, Ott DVM, von Cramon DY. Creative cognition and the brain: Dissociations between frontal, parietal–temporal and basal ganglia groups. *Brain Research*. 2012;1482:55–70. doi:10.1016/j.brainres.2012.09.007.
[20] Ulugut H, Trieu C, Groot C, et al. Overlap of Neuroanatomical Involvement in Frontotemporal Dementia and Primary Psychiatric Disorders: A Meta-analysis. *Biological Psychiatry*. 2023;93:820–828. doi:10.1016/j.biopsych.2022.05.028.
[21] Conca F, Esposito V, Giusto G, Cappa SF, Catricalà E. Characterization of the logopenic variant of Primary Progressive Aphasia: A systematic review and meta-analysis. *Ageing Research Reviews*. 2022;82101760. doi:10.1016/j.arr.2022.101760.
[22] Luo C, Hu N, Xiao Y, Zhang W, Gong Q, Lui S. Comparison of Gray Matter Atrophy in Behavioral Variant Frontal Temporal Dementia and Amyotrophic Lateral Sclerosis: A Coordinate- Based Meta-Analysis. *Frontiers in Aging Neuroscience*. 2020;1214. doi:10.3389/fnagi.2020.00014.
[23] Chapleau M, Aldebert J, Montembeault M, Brambati SM. Atrophy in Alzheimer's Disease and Semantic Dementia: An ALE Meta-Analysis of Voxel-Based Morphometry Studies. *Journal of Alzheimer's Disease*. 2016;54:941–955. doi:10.3233/JAD-160382.
[24] Ellis EG, Joutsa J, Morrison-Ham J, et al. Large-scale activation likelihood estimation meta-analysis of parkinsonian disorders. *Brain Communications*. 2023;5(3)fcad172. doi:10.1093/braincomms/fcad172.
[25] Shen D, Cui L, Fang J, Cui B, Li D, Tai H. Voxel-Wise Meta-Analysis of Gray Matter Changes in Amyotrophic Lateral Sclerosis. *Frontiers in Aging Neuroscience*. 2016;864. doi:10.3389/fnagi.2016.00064.
[26] Geser F, Jellinger KA, Fellner L, Wenning GK, Yilmazer-Hanke D, Haybaeck J. Emergent creativity in frontotemporal dementia. *Journal of Neural Transmission*. 2021;128:279–293. doi:10.1007/s00702-021-02325-z.
[27] Friedberg A, Pasquini L, Diggs R, et al. Prevalence, Timing, and Network Localization of Emergent Visual Creativity in Frontotemporal Dementia. *JAMA Neurology*. 2023:E1–E11. e230001. doi:10.1001/jamaneurol.2023.0001.

© 2025 Kutsche J et al. *JAMA Network Open*.

eTable 1.

Studies reporting fMRI activation coordinates for creative tasks (> control tasks), as initially found and analyzed by Brown & Kim (2021)

Domain	Reference	Table	Demographics
**Divergent Thinking**	Abdul Hamid K, Yusoff AN, Rahman S, Osman SS, Azmi NH, Surat S. Cortical Differential Responses During Divergent Thinking Tasks After Creativity Stimulation. *Psychology & Neuroscience*. 2019;12(3):342–362. doi:10.1037/pne0000168.	2	n=26, mean age: 21.36 (SD 0.59)
**Divergent Thinking**	Abraham A, Pieritz K, Thybusch K, et al. Creativity and the brain: Uncovering the neural signature of conceptual expansion. *Neuropsychologia*. 2012;50:1906–1917. doi:10.1016/j.neuropsychologia.2012.04.015.	2	n=19 (8 male), mean age: 22.42
**Divergent Thinking**	Abraham A, Rutter B, Bantin T, Hermann C. Creative conceptual expansion: A combined fMRI replication and extension T study to examine individual differences in creativity. *Neuropsychologia*. 2018;118:29–39. doi:10.1016/j.neuropsychologia.2018.05.004.	4	n=34 (0 male), mean age: 22.66 (SD 2.88)
**Divergent Thinking**	Benedek M, Schües T, Beaty RE, et al. To create or to recall original ideas: Brain processes associated with the imagination of novel object uses. *Cortex*. 2018;99:93–102. doi:10.1016/j.cortex.2017.10.024.	1	n=42 (17 male), mean age: 24.31 (SD 4.3)
**Divergent Thinking**	Fink A, Benedek M, Koschutnig K, et al. Training of Verbal Creativity Modulates Brain Activity in Regions Associated with language- and memory-Related Demands. *Human Brain Mapping*. 2015;36:4104–4115. doi:10.1002/hbm.22901.	2	n=24 (13 male),<br>n=29 (14 male), mean age: 24.04 (SD 2.93)
**Divergent Thinking**	Fink A, Grabner RH, Benedek M, et al. The Creative Brain: Investigation of Brain Activity During Creative Problem Solving by Means of EEG and fMRI. *Human Brain Mapping*. 2009;30:734–748. doi:10.1002/hbm.20538.	1	n=21 (10 male), mean age: 24.29 (SD 4.09)
**Divergent Thinking**	Fink A, Grabner RH, Gebauer D, Reishofer G, Koschutnig K, Ebner F. Enhancing creativity by means of cognitive stimulation: Evidence from an fMRI study. NeuroImage. 2010;52:1687–1695. doi:10.1016/j.neuroimage.2010.05.072	1	n=31 (13 male), mean age: 23.19 (SD 2.79)
**Divergent Thinking**	Heinonen J, Numminen J, Hlushchuk Y, Antell H, Taatila V, Suomala J. Default Mode and Executive Networks Areas: Association with the Serial Order in Divergent Thinking. *PLOS ONE*. 2016;11(9)e0162234. doi:10.1371/journal.pone.0162234.	1	n=16 (4 male), mean age: 31.3
**Divergent Thinking**	Ivancovsky T, Kleinmintz O, Lee J, Kurman J, Shamay-Tsoory SG. The neural underpinnings of cross-cultural differences in creativity. *Human Brain Mapping*. 2018;39:4493–4508. doi:10.1002/hbm.24288.	2	n=36 (final gender ratio and mean age not reported)
**Divergent Thinking**	Madore KP, Thakral PP, Beaty RE, Addis DR, Schacter DL. Neural Mechanisms of Episodic Retrieval Support Divergent Creative Thinking. *Cerebral Cortex*. 2019;29:150–166. doi:10.1093/cercor/bhx312.	3	n=32 (9 male), mean age: 20.97 (SD 3.11)
**Divergent Thinking**	Mayseless N, Eran A, Shamay-Tsoory SG. Generating original ideas: The neural underpinning of originality. *NeuroImage*. 2015;116:232–239. doi:10.1016/j.neuroimage.2015.05.030.	1	n=25 (final gender ratio and mean age not reported)

© 2025 Kutsche J et al. *JAMA Network Open*.

Domain	Reference	Table	Demographics
**Divergent Thinking**	Sun J, Shi L, Chen Q, et al. Openness to experience and psychophysiological interaction patterns during divergent thinking. *Brain Imaging and Behavior*. 2019;13:1580–1589. doi:10.1007/s11682-018-9965-2.	1	n=29 (12 male), mean age: 19.48 (SD 0.74)
**Divergent Thinking**	Sun J, Chen Q et al. Training your brain to be more creative: brain functional and structural changes induced by divergent thinking training. *Human Brain Mapping*. 2016;37:3375–3387. doi:10.1002/hbm.23246.	3	n=28 (5 male), mean age: 22.39 (SD 2.1)
**Divergent Thinking**	Vartanian O, Beatty EL, Smith I, Blackler K, Lam Q, Forbes S. One-way traffic: The inferior frontal gyrus controls brain activation in the middle temporal gyrus and inferior parietal lobule during divergent thinking. *Neuropsychologia*. 2018;118:68–78. doi:10.1016/j.neuropsychologia.2018.02.024.	1	n=44 (31 male), mean age: 35.47 (SD 11.3)
**Divergent Thinking**	Vartanian O, Bouak F, Caldwell JL, et al. The effects of a single night of sleep deprivation on fluency and prefrontal cortex function during divergent thinking. *Frontiers in Human Neuroscience*. 2014;8214. doi:10.3389/fnhum.2014.00214.	1	n=13 (10 male), mean age: 32.23 (SD 8.45)
**Music**	Bengtsson SL, Csíkszentmihályi M, Ullén F. Cortical Regions Involved in the Generation of Musical Structures during Improvisation in Pianists. *Journal of Cognitive Neuroscience*. 2007;19(5):830–842. doi:10.1162/jocn.2007.19.5.830.	2	n=11 (11 male), mean age: 32.0 (SD 6.0)
**Music**	Berkowitz AL, Ansari D. Generation of novel motor sequences: The neural correlates of musical improvisation. *NeuroImage*. 2008;41:535–543. doi:10.1016/j.neuroimage.2008.02.028.	2	n=13 (5 male), mean age: 21.9
**Music**	de Aquino MPB, Verdejo-Román J, Pérez-García M, Pérez-García P. Different role of the supplementary motor area and the insula between musicians and non-musicians in a controlled musical creativity task. *Scientific Reports*. 2019;913006. doi:10.1038/s41598-019-49405-5.	3	n=19 (9 male), mean age: 20.26 (SD 2.05)
**Music**	de Manzano Ö, Ullén F. Goal-independent mechanisms for free response generation: Creative and pseudo-random performance share neural substrates. *NeuroImage*. 2012;59:772–780. doi:10.1016/j.neuroimage.2011.07.016.	1	n=18 (17 male), mean age: 39 (SD 12)
**Music**	de Manzano Ö, Ullén F. Activation and connectivity patterns of the presupplementary and dorsal premotor areas during free improvisation of melodies and rhythms. *NeuroImage*. 2012;63:272–280. doi:10.1016/j.neuroimage.2012.06.024.	S1	n=15 (14 male), mean age: 40 (SD 12)
**Music**	Dhakal K, Norgaard M, Adhikari BM, Yun KS, Dhamala M. Higher Node Activity with Less Functional Connectivity During Musical Improvisation. *Brain Connectivity*. 2019;20(20):1–14. doi:10.1089/brain.2017.0566.	2	n=24 (24 male), mean age: 31.9 (SD 13.6)
**Music**	Donnay GF, Rankin SK, Lopez-Gonzalez M, Jiradejvong P, Limb CJ. Neural Substrates of Interactive Musical Improvisation: An fMRI Study of 'Trading Fours' in Jazz. *PLOS ONE*. 2014;9(2)e88665. doi:10.1371/journal.pone.0088665.	2	n=11 (11 male), mean age: 38.8 SD 11)
**Music**	Limb CJ, Braun AR. Neural Substrates of Spontaneous Musical Performance: An fMRI Study of Jazz Improvisation. *PLOS ONE*. 2008;3(2)e1679. doi:10.1371/journal.pone.0001679.	2, 3	n=6 (6 male), mean age: 34.2 (SD 10.4)

© 2025 Kutsche J et al. *JAMA Network Open*.

Domain	Reference	Table	Demographics
**Music**	McPherson MJ, Barrett FS, Lopez-Gonzalez M, Jiradejvong P, Limb CJ. Emotional Intent Modulates The Neural Substrates Of Creativity: An fMRI Study of Emotionally Targeted Improvisation in Jazz Musicians. *Scientific Reports*. 2016;618460. doi:10.1038/srep18460.	1	n=12 (11 male), mean age: 39.9 (SD 15.8)
**Music**	Villarreal MF, Cerquetti D, Caruso S, et al. Neural Correlates of Musical Creativity: Differences between High and Low Creative Subjects. *PLOS ONE*. 2013;8(9)e75427. doi:10.1371/journal.pone.0075427.	2	n=12 (final gender ratio and mean age not reported)
**Drawing**	Ellamil M, Dobson C, Beeman M, Christoff K. Evaluative and generative modes of thought during the creative process. *NeuroImage*. 2012;59:1783–1794. doi:10.1016/j.neuroimage.2011.08.008.	1	n=15 (6 male), mean age: 22.14 (SD 2.25)
**Drawing**	Hahm J, Kim KK, Park S-H, Lee H-M. Brain Areas Subserving Torrance Tests of Creative Thinking: An Functional Magnetic Resonance Imaging Study. *Dementia and Neurocognitive Disorders*. 2017;16(2):48–53. doi:10.12779/dnd.2017.16.2.48.	2	n=25 (11 male), mean age: 19.9 (SD 1.8)
**Drawing**	Park HRP, Kirk IJ, Waldie KE. Neural correlates of creative thinking and schizotypy. *Neuropsychologia*. 2015;73:94–107. doi:10.1016/j.neuropsychologia.2015.05.007.	1	n=48 (17 male), mean age: 23.42 (SD 4.50)
**Drawing**	Saggar M, Quintin E-M, Kienitz E, et al. Pictionary-based fMRI paradigm to study the neural correlates of spontaneous improvisation and figural creativity. *Scientific Reports*. 2015;510894. doi:10.1038/srep10894.	1	n=30 (14 male), mean age: 28.77 (SD 5.54)
**Writing**	Liu S, Erkkinen MG, Healey ML, et al. Brain Activity and Connectivity During Poetry Composition: Toward a Multidimensional Model of the Creative Process. *Human Brain Mapping*. 2015;36:3351–3372. doi:10.1002/hbm.22849.	S3	n=27 (12 male), mean age: 31.62 (SD 10.76)
**Writing**	Shah C, Erhard K, Ortheil H-J, Kaza E, Kessler C, Lotze M. Neural Correlates of Creative Writing: An fMRI Study. *Human Brain Mapping*. 2013;34:1088–1101. doi:10.1002/hbm.21493.	3	n=28 (14 male), mean age: 24.0 (SD 1.9)
**Dance**	Chauvigné LAS, Belyk M, Brown S. Taking two to tango: fMRI analysis of improvised joint action with physical contact. *PLOS ONE*. 2018;13(1)e0191098. doi:10.1371/journal.pone.0191098.	3	n=18 (9 male), mean age: 40.4 (SD 13.2)
**Verbalization**	Amir O, Biederman I. The Neural Correlates of Humor Creativity. *Frontiers in Human Neuroscience*. 2016;10597. doi:10.3389/fnhum.2016.00597.	1	n=13 (12 male), mean age: 35.4<br>n=9 (7 male) mean age: 27.2<br>n=18 (11 male), mean age: 24.9
**Verbalization**	Bechtereva NP, Korotkov AD, Pakhomov SV, Roudas MS, Starchenko MG, Medvedev SV. PET study of brain maintenance of verbal creative activity. *International Journal of Psychophysiology*. 2004;53:11–20. doi:10.1016/j.ijpsycho.2004.01.001.	1	n=16 (16 male), mean age not reported
**Verbalization**	Howard-Jones PA, Blakemore S-J, Samuel EA, Summers IR, Claxton G. Semantic divergence and creative story	4	n=8 (1 male), mean age: 21.9, (SD 2.7)

© 2025 Kutsche J et al. *JAMA Network Open*.

Domain	Reference	Table	Demographics
	generation: An fMRI investigation. *Cognitive Brain Research*. 2005;25:240–250. doi:10.1016/j.cogbrainres.2005.05.013.		
**Verbalization**	Liu S, Chow HM, Xu Y, et al. Neural Correlates of Lyrical Improvisation: An fMRI Study of Freestyle Rap. *Scientific Reports*. 2012;2834. doi:10.1038/srep00834.	1	n=12 (12 male), mean age: 30.3

© 2025 Kutsche J et al. *JAMA Network Open*.

eFigure 1.

The topography of the network overlap map is robust across different t-thresholds and sphere sizes

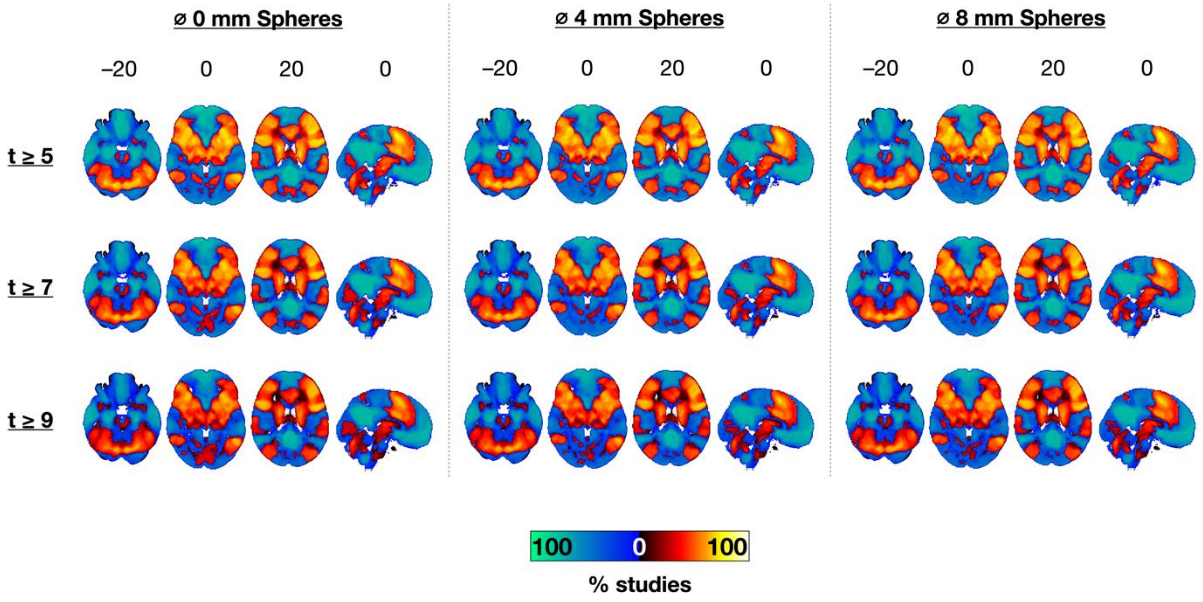

© 2025 Kutsche J et al. *JAMA Network Open*.

eTable 2.

Spatial Pearson correlations between network overlap maps for the *t*-thresholds 5, 7, and 9 and across different sphere sizes (0 mm, 4 mm, 8 mm)

		t ≥ 5			t ≥ 7			t ≥ 9		
		**0 mm**	**4 mm**	**8 mm**	**0 mm**	**4 mm**	**8 mm**	**0 mm**	**4 mm**	**8 mm**
**t ≥ 5**	**0 mm**		0.96	0.96	0.95	0.95	0.95	0.92	0.92	0.92
	**4 mm**	0.96		0.98	0.94	0.95	0.96	0.91	0.92	0.92
	**8 mm**	0.96	0.98		0.94	0.95	0.95	0.91	0.91	0.92
**t ≥ 7**	**0 mm**	0.95	0.94	0.94		0.96	0.96	0.95	0.95	0.95
	**4 mm**	0.95	0.95	0.95	0.96		0.98	0.94	0.95	0.96
	**8 mm**	0.95	0.96	0.95	0.96	0.98		0.93	0.94	0.95
**t ≥ 9**	**0 mm**	0.92	0.91	0.91	0.95	0.94	0.93		0.96	0.96
	**4 mm**	0.92	0.92	0.91	0.95	0.95	0.94	0.96		0.98
	**8 mm**	0.92	0.92	0.92	0.95	0.96	0.95	0.96	0.98	

© 2025 Kutsche J et al. *JAMA Network Open*.

eFigure 2.
Coordinate Network Mapping (CNM) of creativity-specific activation foci on three different levels

(A) Peak activation coordinates reported in a recent ALE meta-analysis (Kim & Brown, 2021), (B) peak activation coordinates reported in each study that went into the ALE meta-analysis combined to study-level seeds, (C) peak activation coordinates reported in each study individually. Functional connectivity was calculated using a 1000-subject resting-state functional connectome. Positive functional connectivity to the activation foci is shown in warm colors, negative functional connectivity is shown in cool colors. In the study-level network overlap map, 11 of the activation peaks falling within the creativity circuit relate to divergent thinking, while 21 relate to motoric creativity.

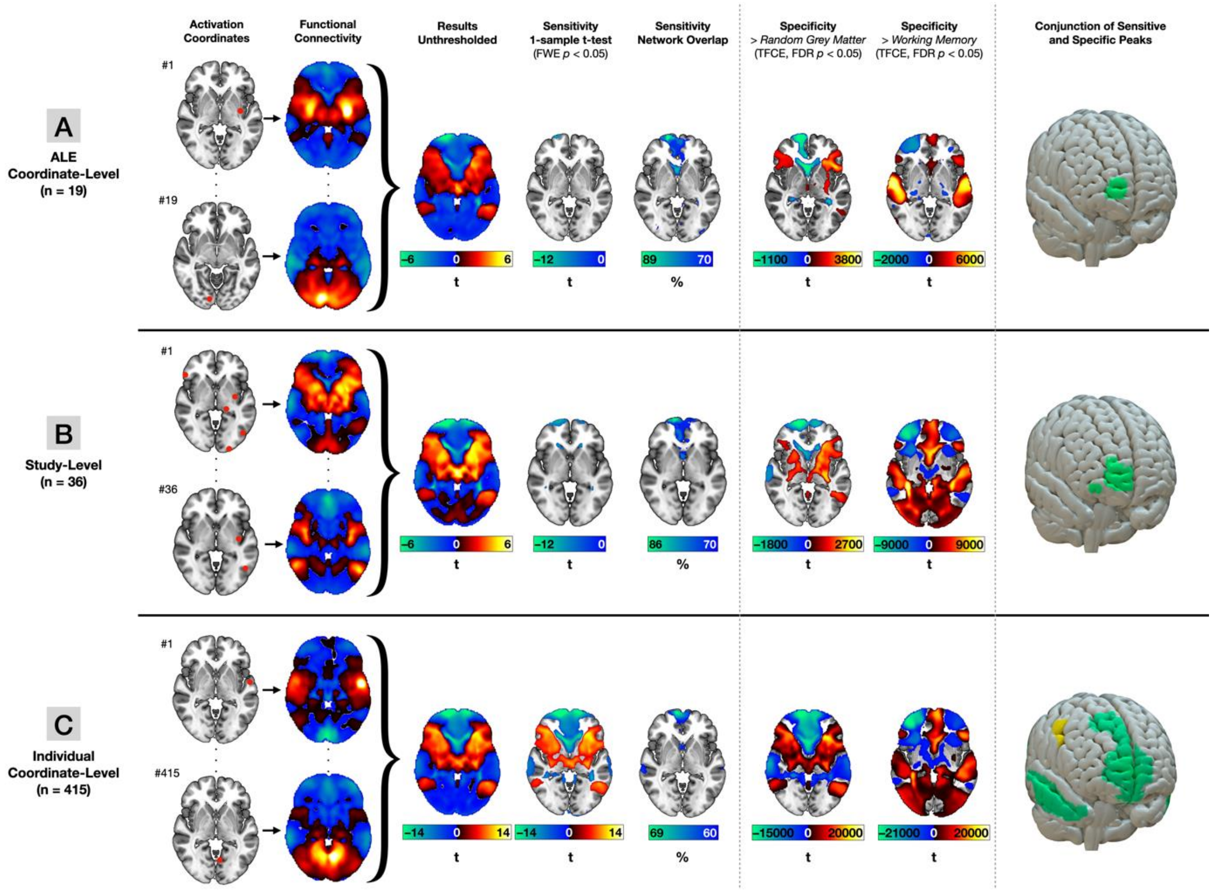

© 2025 Kutsche J et al. *JAMA Network Open*.

eTable 3.
Additional studies reporting fMRI activation coordinates for creative tasks (not controlled for task-based activation) as initially found and analyzed by Chen et al. (2020) and Gonen-Yaacovi et al. (2013)

Reference	Table
Benedek M, Beaty RE, Jauk E, et al. Creating metaphors: The neural basis of figurative language production. *NeuroImage*. 2014;90:99–106. doi:10.1016/j.neuroimage.2013.12.046.	1
Beaty RE, Silvia PJ, Benedek M. Brain networks underlying novel metaphor production. *Brain and Cognition*. 2017;111:163–170. doi:10.1016/j.bandc.2016.12.004.	1
Erhard K, Kessler F, Neumann N, Ortheil H-J, Lotze M. Professional training in creative writing is associated with enhanced fronto-striatal activity in a literary text continuation task. *NeuroImage*. 2014;100:15–23. doi:10.1016/j.neuroimage.2014.05.076.	1
Aziz-Zadeh L, Liew S-L, Dandekar F. Exploring the neural correlates of visual creativity. *SCAN*. 2013;8:475–480. doi:10.1093/scan/nss021.	1
Cai Y, Zhang D, Liang B, et al. Relation of visual creative imagery manipulation to resting-state brain oscillations. *Brain Imaging and Behavior*. 2018;12:258–273. doi:10.1007/s11682-017-9689-8.	1
Fan L, Fan X, Luo W, Wu G, Yan X, Yin D, ... Xu D. An explorative fMRI study of human creative thinking using a specially designed iCAD system. *Acta Psychologica Sinica*. 2014;46(4):427–436. doi:10.3724/SP.J.1041.2014.00427.	1
Huang P, Qiu L, Shen L, et al. Evidence for a Left-Over-Right Inhibitory Mechanism During Figural Creative Thinking in Healthy Nonartists. *Human Brain Mapping*. 2013;34:2724–2732. doi:10.1002/hbm.22093.	1
Kowatari Y, Lee SH, Yamamura H, et al. Neural Networks Involved in Artistic Creativity. *Human Brain Mapping*. 2009;30:1678–1690. doi:10.1002/hbm.20633.	1
Saggar M, Quintin E-M, Bott NT, et al. Changes in Brain Activation Associated with Spontaneous Improvization and Figural Creativity After Design-Thinking-Based Training: A Longitudinal fMRI Study. *Cerebral Cortex*. 2017;27:3542–3552. doi:10.1093/cercor/bhw171.	S1
Pinho AL, Ullén F, Castelo-Branco M, Fransson P, de Manzano Ö. Addressing a Paradox: Dual Strategies for Creative Performance in Introspective and Extrospective Networks. *Cerebral Cortex*. 2016;26:3052–3063. doi:10.1093/cercor/bhv130.	Text
Lu J, Yang H, He H, et al. The Multiple-Demand System in the Novelty of Musical Improvisation: Evidence from an MRI Study on Composers. *Frontiers in Neuroscience*. 2017;11695. doi:10.3389/fnins.2017.00695.	2
Brown S, Martinez MJ, Parsons LM. Music and language side by side in the brain: a PET study of the generation of melodies and sentences. *European Journal of Neuroscience*. 2006;23:2791–2803. doi:10.1111/j.1460-9568.2006.04785.x.	1
Asari T, Konishi S, Jimura K, Chikazoe J, Nakamura N, Miyashita Y. Right temporopolar activation associated with unique perception. *NeuroImage*. 2008;41:145–152. doi:10.1016/j.neuroimage.2008.01.059.	2
Aziz-Zadeh L, Kaplan JT, Iacoboni M. ‘‘Aha!’’: The Neural Correlates of Verbal Insight Solutions. *Human Brain Mapping*. 2009;30:908–916. doi:10.1002/hbm.20554.	1
Cardillo ER, Watson CE, Schmidt GL, Kranjec A, Chatterjee A. From novel to familiar: Tuning the brain for metaphors. *NeuroImage*. 2012;59:3212–3221. doi:10.1016/j.neuroimage.2011.11.079.	3
Chrysikou EG, Thompson-Schill SL. Dissociable Brain States Linked to Common and Creative Object Use. *Human Brain Mapping*. 2011;32(32):665–675. doi:10.1002/hbm.21056.	Text
Geake JG, Hansen PC. Neural correlates of intelligence as revealed by fMRI of fluid analogies. *NeuroImage*. 2005;26:555–564. doi:10.1016/j.neuroimage.2005.01.035.	2
Goel V, Vartanian O. Dissociating the Roles of Right Ventral Lateral and Dorsal Lateral Prefrontal Cortex in Generation and Maintenance of Hypotheses in Set-shift Problems. *Cerebral Cortex*. 2005;15:1170–1177. doi:10.1093/cercor/bhh217.	1
Green AE, Kraemer DJM, Fugelsang JA, Gray JR, Dunbar KN. Neural Correlates of Creativity in Analogical Reasoning. *Journal of Experimental Psychology: Learning, Memory, and Cognition*. 2012;38(2):264–272. doi:10.1037/a0025764.	1, 2
Jung-Beeman M, Bowden EM, Haberman J, et al. Neural Activity When People Solve Verbal Problems with Insight. *PLOS Biology*. 2004;2(4):0500–0510. doi:10.1371/journal.pbio.0020097.	1
Kounios J, Frymiare JL, Bowden EM, et al. The Prepared Mind: Neural Activity Prior to Problem Presentation Predicts Subsequent Solution by Sudden Insight. *Psychological Science*. 2006;17(10):882–890. doi:10.1111/j.1467-9280.2006.01798.x.	1
Kröger S, Rutter B, Stark R, Windmann S, Hermann C, Abraham A. Using a shoe as a plant pot: Neural correlates of passive conceptual expansion. *Brain Research*. 2012;1430:52–61. doi:10.1016/j.brainres.2011.10.031.	2
Luo J, Niki K, Phillips S. Neural correlates of the ‘Aha! reaction’. *NeuroReport*. 2004;15(13):2013–2017. doi:10.1097/00001756-200409150-00004.	1
Mashal N, Faust M, Hendler T, Jung-Beeman M. An fMRI investigation of the neural correlates underlying the processing of novel metaphoric expressions. *Brain and Language*. 2007;100:115–126. doi:10.1016/j.bandl.2005.10.005.	1
Qiu J, Li H, Jou J, et al. Neural correlates of the ‘‘Aha’’ experiences: Evidence from an fMRI study of insight problem solving. *Cortex*. 2010;46:397–403. doi:10.1016/j.cortex.2009.06.006.	1
Rutter B, Kröger S, Stark R, et al. Can clouds dance? Neural correlates of passive conceptual expansion using a metaphor processing task: Implications for creative cognition. *Brain and Cognition*. 2012;78:114–122. doi:10.1016/j.bandc.2011.11.002.	3

© 2025 Kutsche J et al. *JAMA Network Open*.

Reference	Table
Seeger CA, Desmond JE, Glover GH, Gabrieli JDE. Functional Magnetic Resonance Imaging Evidence for Right-Hemisphere Involvement in Processing Unusual Semantic Relationship. *Neuropsychology*. 2000;14(3):361–369. doi:10.1037//0894-4105.14.3.361.	2
Siebörger FT, Ferstl EC, von Cramon DY. Making sense of nonsense: An fMRI study of task induced inference processes during discourse comprehension. *Brain Research*. 2007;1166:77–91. doi:10.1016/j.brainres.2007.05.079.	4
Tian F, Tu S, Qiu J, Lv JY, Wei DT, Su YH, Zhang QL. Neural correlates of mental preparation for successful insight problem solving. *Behavioural Brain Research*. 2011;216:626–630. doi:10.1016/j.bbr.2010.09.005.	1
Vartanian O, Goel V. Task constraints modulate activation in right ventral lateral prefrontal cortex. *NeuroImage*. 2005;27:927–933. doi:10.1016/j.neuroimage.2005.05.016.	2

© 2025 Kutsche J et al. *JAMA Network Open*.

eTable 4

Studies reporting fMRI activation coordinates for working memory as initially found by Rottschy et al. (2012) and Wang et al. (2019)

Reference	Table
Allen PP, Cleare AJ, Lee F et al. Effect of acute tryptophan depletion on pre-frontal engagement. *Psychopharmacology (Berl)*. 2006;187(4):486–497. doi:10.1007/s00213-006-0444-x.	2
Altamura M, Elvevåg B, Blasi G et al. Dissociating the effects of Sternberg working memory demands in prefrontal cortex. *Psychiatry Research*. 2007;154(2):103–114. doi:10.1016/j.pscychresns.2006.08.002.	3, 4
Audoin B, Duong MVA, Ranjeva JP et al. Magnetic resonance study of the influence of tissue damage and cortical reorganization on PASAT performance at the earliest stage of multiple sclerosis. *Human Brain Mapping*. 2005;24(3):216–228. doi:10.1002/hbm.20083.	4
Axmacher N, Haupt S, Cohen M et al. Interference of working memory load with long-term memory formation. *European Journal of Neuroscience*. 2009;29(7):1501–1513. doi:10.1111/j.1460-9568.2009.06676.x.	1
Axmacher N, Mormann F, Fernández G et al. Sustained Neural Activity Patterns during Working Memory in the Human Medial Temporal Lobe. *Journal of Neuroscience*. 2007;28(29):7807–7816. doi:10.1523/JNEUROSCI.0962-07.2007.	1
Bedwell JS, Horner MD, Yamanaka K et al. Functional neuroanatomy of subcomponent cognitive processes involved in verbal working memory. *International Journal of Neuroscience*. 2005;115(7):1017–1032. doi:10.1080/00207450590901530.	1
Belayachi S, Majerus S, Gendolla G et al. Are the carrot and the stick the two sides of same coin? A neural examination of approach/avoidance motivation during cognitive performance. *Behavioural Brain Research*. 2015;293:217–226. doi:10.1016/j.bbr.2015.07.042.	2
Beneventi H, Barndon R, Ersland L, Hugdahl K. An fMRI study of working memory for schematic facial expressions. *Scandinavian Journal of Psychology*. 2007;48:81–86. doi:10.1111/j.1467-9450.2007.00536.x.	1
Berger C, Erbe AK, Ehlers I et al. Effects of task-irrelevant emotional stimuli on working memory processes in mild cognitive impairment. *Journal of Alzheimer's Disease*. 2015;44(2):439–453. doi:10.3233/JAD-141848.	4
Binder M, Urbanik AS. Material-dependent activation in prefrontal cortex: working memory for letters and texture patterns--initial observations. *Radiology*. 2006;238(1):256–263. doi:10.1148/radiol.2381041622.	2
Bunge SA, Ochsner KN, Desmond JE, Glover GH, Gabrieli JH. Prefrontal regions involved in keeping information in and out of mind. *Brain*. 2001;124(Pt 10):2074–2086. doi:10.1093/brain/124.10.2074.	1
Cader S, Cifelli A, Abu-Omar Y, Palace J, Matthews PM. Reduced brain functional reserve and altered functional connectivity in patients with multiple sclerosis. *Brain*. 2006;129(2):527–537. doi:10.1093/brain/awh670.	2
Cairo TA, Liddle PF, Woodward TS, Ngan ETC. The influence of working memory load on phase specific patterns of cortical activity. *Cognitive Brain Research*. 2004;21(3):377–387. doi:10.1016/j.cogbrainres.2004.06.014.	1, 2, 3
Caldú X, Vendrell P, Bartrés-Faz D et al. Impact of the COMT Val108/158 Met and DAT genotypes on prefrontal function in healthy subjects. *NeuroImage*. 2007;37(4):1437–1444. doi:10.1016/j.neuroimage.2007.06.021.	2
Caldwell JA, Mu Q, Smith JK et al. Are individual differences in fatigue vulnerability related to baseline differences in cortical activation?. *Behavioral Neuroscience*. 2005;119(3):694–707. doi:10.1037/0735-7044.119.3.694.	1

© 2025 Kutsche J et al. *JAMA Network Open*.

Reference	Table
Callicott JH, Mattay VS, Bertolino A et al. Physiological characteristics of capacity constraints in working memory as revealed by functional MRI. *Cerebral Cortex*. 1999;9(1):20–26. doi:10.1093/cercor/9.1.20.	1
Camchong J, Dyckman KA, Chapman CE et al. Basal ganglia-thalamocortical circuitry disruptions in schizophrenia during delayed response tasks. *Biological Psychiatry*. 2006;60(3):235–241. doi:10.1016/j.biopsych.2005.11.014.	1
Caseras X, Mataix-Cols D, Giampietro V et al. Probing the working memory system in chronic fatigue syndrome: a functional magnetic resonance imaging study using the n-back task. *Psychosomatic Medicine*. 2006;68(6):947–955. doi:10.1097/01.psy.0000242770.50979.5f.	2
Cerasa A, Gioia MC, Fera F et al. Ventro-lateral prefrontal activity during working memory is modulated by MAO A genetic variation. *Brain Research*. 2008;1201:114–121. doi:10.1016/j.brainres.2008.01.048.	2
Choo WC, Lee WW, Venkatraman V et al. Dissociation of cortical regions modulated by both working memory load and sleep deprivation and by sleep deprivation alone. *NeuroImage*. 2005;25(2):579–587. doi:10.1016/j.neuroimage.2004.11.029.	2
Christodoulou C, DeLuca J, Ricker JH et al. Functional magnetic resonance imaging of working memory impairment after traumatic brain injury. *Journal of Neurology, Neurosurgery & Psychiatry*. 2001;71(2):161–168. doi:10.1136/jnnp.71.2.161.	2
Ciesielski KT, Lesnik PG, Savoy RL, Grant EP, Ahlfors SP. Developmental neural networks in children performing a Categorical N-Back Task. *NeuroImage*. 2006;33(3):980–990. doi:10.1016/j.neuroimage.2006.07.028.	2
Cohen JD, Perlstein WM, Braver TS et al. Temporal dynamics of brain activation during a working memory task. *Nature*. 1997;386(6625):604–608. doi:10.1038/386604a0.	1
Cross ES, Schmitt PJ, Grafton ST. Neural substrates of contextual interference during motor learning support a model of active preparation. *Journal of Cognitive Neuroscience*. 2007;19(11):1854–1871. doi:10.1162/jocn.2007.19.11.1854.	1, 2
Deckersbach T, Rauch SL, Buhlmann U et al. An fMRI investigation of working memory and sadness in females with bipolar disorder: a brief report. *Bipolar Disorders*. 2008;10(8):928–942. doi:10.1111/j.1399-5618.2008.00633.x.	3
Derrfuss J, Brass M, von Cramon DY. Cognitive control in the posterior frontolateral cortex: evidence from common activations in task coordination, interference control, and working memory. *NeuroImage*. 2004:23(2):604–612. doi:10.1016/j.neuroimage.2004.06.007.	1, A-B
Desmond JE, Chen SHA, DeRosa E et al. Increased frontocerebellar activation in alcoholics during verbal working memory: an fMRI study. *NeuroImage*. 2003;19(4):1510–1520. doi:10.1016/s1053-8119(03)00102-2.	3
Dima D, Jogia J, Frangou S. Dynamic causal modeling of load-dependent modulation of effective connectivity within the verbal working memory network. *Human Brain Mapping*. 2014;35(7):3025–3035. doi:10.1002/hbm.22382.	2
Döhnel K, Sommer M, Ibach B et al. Neural correlates of emotional working memory in patients with mild cognitive impairment. *Neuropsychologia*. 2008;46(1):37–48. doi:10.1016/j.neuropsychologia.2007.08.012.	5
Drapier D, Surguladze S, Marshall N et al. Genetic liability for bipolar disorder is characterized by excess frontal activation in response to a working memory task. *Biological Psychiatry*. 2008;64(6):513–520. doi:10.1016/j.biopsych.2008.04.038.	2
Drobyshevsky A, Baumann SB, Schneider W. A rapid fMRI task battery for mapping of visual, motor, cognitive, and emotional function. *NeuroImage*. 2006;31(2):732–744. doi:10.1016/j.neuroimage.2005.12.016.	2
Druzgal TJ, D'Esposito M. Activity in fusiform face area modulated as a function of working memory load. *Cognitive Brain Research*. 2001;10(3):355–364. doi:10.1016/S0926-6410(00)00056-2.	3

Reference	Table
Duggirala SX, Saharan S, Raghunathan P, Mandal PK. Stimulus-dependent modulation of working memory for identity monitoring: A functional MRI study. *Brain and Cognition*. 2016;102:55–64. doi:10.1016/j.bandc.2015.12.006.	2
El-Hage W, Phillips ML, Radua J et al. Genetic modulation of neural response during working memory in healthy individuals: interaction of glucocorticoid receptor and dopaminergic genes. *Molecular Psychiatry*. 2013;18:174–182. doi:10.1038/mp.2011.145.	2
Elliott R, Dolan RJ. Differential Neural Responses during Performance of Matching and Nonmatching to Sample Tasks at Two Delay Intervals. *Journal of Neuroscience*. 1999;19(12):5066–5073. doi:10.1523/JNEUROSCI.19-12-05066.1999.	2
Elzinga BM, Ardon AM, Heijnis MK et al. Neural correlates of enhanced working-memory performance in dissociative disorder: a functional MRI study. *Psychological Medicine*. 2007;37(2):235–245. doi:10.1017/S0033291706008932.	3
Engström M, Vigren P, Karlsson T, Landtblom AM. Working Memory in 8 Kleine-Levin Syndrome Patients: An fMRI Study. *Sleep.* 2009;32(5):681–688. doi:10.1093/sleep/32.5.681.	5
Fernández-Corcuera P, Salvador R, Monté GC et al. Bipolar depressed patients show both failure to activate and failure to de-activate during performance of a working memory task. *Journal of Affective Disorders*. 2013;148(2–3):170–178. doi:10.1016/j.jad.2012.04.009.	2
Forn C, Barros-Loscertales A, Escudero J et al. Cortical reorganization during PASAT task in MS patients with preserved working memory functions. *NeuroImage*. 2006;31(2):686–691. doi:10.1016/j.neuroimage.2005.12.030.	3
Forn C, Barros-Loscertales A, Escudero J et al. Compensatory activations in patients with multiple sclerosis during preserved performance on the auditory N-back task. *Human Brain Mapping*. 2007;28(5):424–430. doi:10.1002/hbm.20284.	2
Frangou S, Kington J, Raymont V, Shergill SS. Examining ventral and dorsal prefrontal function in bipolar disorder: a functional magnetic resonance imaging study. *European Psychiatry*. 2008;23(4):300–308. doi:10.1016/j.eurpsy.2007.05.002.	1, 2
Garavan H, Kelley D, Rosen A, Rao SM, Stein EA. Practice-related functional activation changes in a working memory task. 2000. *Microscopy Research & Technique*. 2000;51(1):54–63. doi:10.1002/1097-0029(20001001)51:1<54::AID-JEMT6>3.0.CO;2-J.	1
Garavan H, Ross TJ, Li SJ, Stein EA. A parametric manipulation of central executive functioning. *Cerebral Cortex*. 2000;10(6):585–592. doi:10.1093/cercor/10.6.585.	2
Garraux G, Hallett M, Talagala SL. CASL fMRI of subcortico-cortical perfusion changes during memory-guided finger sequences. *NeuroImage*. 2005;25(1):122–132. doi:10.1016/j.neuroimage.2004.11.004.	1
Garrett A, Kelly R, Gomez R et al. Aberrant Brain Activation During a Working Memory Task in Psychotic Major Depression. *American Journal of Psychiatry*. 2011;168(2):173–182. doi:10.1176/appi.ajp.2010.09121718.	2
Goldstein JM, Jerram M, Poldrack R et al. Sex differences in prefrontal cortical brain activity during fMRI of auditory verbal working memory. *Neuropsychology*. 2005;19(4):509–519. doi:10.1037/0894-4105.19.4.509.	3
Gropman AL, Shattuck K, Prust MJ et al. Altered neural activation in ornithine transcarbamylase deficiency during executive cognition: an fMRI study. *Human Brain Mapping*. 2013;34(4):753–761. doi:10.1002/hbm.21470.	2
Grosbas MH, Leonards U, Lobel E et al. Human Cortical Networks for New and Familiar Sequences of Saccades. *Cerebral Cortex*. 2001;10:936–945. doi:10.1093/cercor/11.10.936.	1
Gruber O, Tost H, Henseler I et al. Pathological amygdala activation during working memory performance: Evidence for a pathophysiological trait marker in bipolar affective disorder. *Human Brain Mapping*. 2010;31(1):115–125. doi:10.1002/hbm.20849.	2

© 2025 Kutsche J et al. *JAMA Network Open*.

Reference	Table
Harvey PO, Fossati P, Pochon JB et al. Cognitive control and brain resources in major depression: an fMRI study using the n-back task. *NeuroImage*. 2005;26(3):860–869. doi:10.1016/j.neuroimage.2005.02.048.	2
Honey GD, Bullmore ET, Sharma T. Prolonged reaction time to a verbal working memory task predicts increased power of posterior parietal cortical activation. *NeuroImage*. 2000;12(5):495–503. doi:10.1006/nimg.2000.0624.	1
Honey GD, Sharma T, Suckling J et al. The functional neuroanatomy of schizophrenic subsyndromes. *Psychological Medicine*. 2003;33(6):1007–1018. doi:10.1017/s0033291703007864.	3
Huang AS, Klein DN, Leung HC. Load-related brain activation predicts spatial working memory performance in youth aged 9-12 and is associated with executive function at earlier ages. *Developmental Cognitive Neuroscience*. 2016;17:1–9. doi:10.1016/j.dcn.2015.10.007.	1
Johnson MR, Morris NA, Astur RS et al. A functional magnetic resonance imaging study of working memory abnormalities in schizophrenia. *Biological Psychiatry*. 2006;60(1):11–21. doi:10.1016/j.biopsych.2005.11.012.	6, 7
Kaas AL, van Mier H, Goebel R. The Neural Correlates of Human Working Memory for Haptically Explored Object Orientations. *Cerebral Cortex*. 2007;17(7):1637–1649. doi:10.1093/cercor/bhl074.	5
Kim J, Whyte J, Wang J et al. Continuous ASL perfusion fMRI investigation of higher cognition: quantification of tonic CBF changes during sustained attention and working memory tasks. *NeuroImage*. 2006;31(1):376–385. doi:10.1016/j.neuroimage.2005.11.035.	2
Knops A, Nuerk HC, Fimm B, Vohn R, Willmes K. A special role for numbers in working memory? An fMRI study. *NeuroImage*. 2006;29(1):1–14. doi:10.1016/j.neuroimage.2005.07.009.	1
Koelsch S, Schulze K, Sammler D et al. Functional architecture of verbal and tonal working memory: An FMRI study. *Human Brain Mapping*. 2009;30(3):859–873. doi:10.1002/hbm.20550.	1
Koppelstaetter F, Poeppel TD, Siedentopf CM et al. Does caffeine modulate verbal working memory processes? An fMRI study. *NeuroImage*. 2008;39(1):492–499. doi:10.1016/j.neuroimage.2007.08.037.	2
Koshino H, Kana RK, Keller TA et al. fMRI investigation of working memory for faces in autism: visual coding and underconnectivity with frontal areas. *Cerebral Cortex*. 2008;18(2):289–300. doi:10.1093/cercor/bhm054.	3
Kumari V, Aasen I, Taylor P et al. Neural dysfunction and violence in schizophrenia: an fMRI investigation. *Schizophrenia Researc*. 2006;84(1):144–164. doi:10.1016/j.schres.2006.02.017.	2
Kumari V, Gray JA, ffytche DH et al. Cognitive effects of nicotine in humans: an fMRI study. *NeuroImage*. 2003;19(3):1002–1013. doi:10.1016/s1053-8119(03)00110-1.	1
LaBar KS, Gitelman DR, Parrish TB, Mesulam M. Neuroanatomic overlap of working memory and spatial attention networks: a functional MRI comparison within subjects. *NeuroImage*. 1999;10(6):695–704. doi:10.1006/nimg.1999.0503.	3
Lagopoulos J, Ivanovski B, Malhi GS. An event-related functional MRI study of working memory in euthymic bipolar disorder. *Journal of Psychiatry & Neuroscience*. 2007;32(3):174–184.	2, 3, 4
Lamp G, Alexander B, Laycock R, Crewther DP, Crewther SG. Mapping of the Underlying Neural Mechanisms of Maintenance and Manipulation in Visuo-Spatial Working Memory Using An n-back Mental Rotation Task: A Functional Magnetic Resonance Imaging Study. *Froniers in Behavioral Neuroscience*. 2016;10:87. doi:10.3389/fnbeh.2016.00087.	2
Landau SM, Lal R, O'Neil JP, Baker S, Jagust WJ. Striatal Dopamine and Working Memory. *Cerebral Cortex*. 2009;19(2):445–454. doi:10.1093/cercor/bhn095.	2

© 2025 Kutsche J et al. *JAMA Network Open*.

Reference	Table
Landau SM, Schumacher EH, Garavan H, Druzgal TJ, D'Esposito M. A functional MRI study of the influence of practice on component processes of working memory. *NeuroImage*. 2004;22(1):211–221. doi:10.1016/j.neuroimage.2004.01.003.	1
Lee TW, Liu HL, Wai YY, Ko HJ, Lee SH. Abnormal neural activity in partially remitted late-onset depression: an fMRI study of one-back working memory task. *Psychiatry Research*. 2013;213(2)133–142. doi:10.1016/j.pscychresns.2012.04.010.	2
Lim HK, Juh R, Pae CU et al. Altered verbal working memory process in patients with Alzheimer's disease: an fMRI investigation. *Neuropsychobiology*. 2008;57(4):181–187. doi:10.1159/000147471.	2
Linden DEJ, Bittner RA, Muckli L et al. Cortical capacity constraints for visual working memory: dissociation of fMRI load effects in a fronto-parietal network. *NeuroImage*. 2003;20(3):1518–1530. doi:10.1016/j.neuroimage.2003.07.021.	2
LoPresti ML, Schon K, Tricarico MD et al. Working Memory for Social Cues Recruits Orbitofrontal Cortex and Amygdala: A Functional Magnetic Resonance Imaging Study of Delayed Matching to Sample for Emotional Expressions. *Journal of Neuroscience.* 2008;28(14):3718–3728. doi:10.1523/JNEUROSCI.0464-08.2008.	1
Loughead J, Wileyto EP, Valdez JN et al. Effect of abstinence challenge on brain function and cognition in smokers differs by COMT genotype. *Molecular Psychiatry*. 2009;14:820–826. doi:10.1038/mp.2008.132.	2
Luo Y, Qin S, Fernández G et al. Emotion perception and executive control interact in the salience network during emotionally charged working memory processing. *Human Brain Mapping*. 2014;35(11):5606–5616. doi:10.1002/hbm.22573.	1
Lythe KE, Williams SCR, Anderson C, Libri V, Mehta MA. Frontal and parietal activity after sleep deprivation is dependent on task difficulty and can be predicted by the fMRI response after normal sleep. *Behavioural Brain Research*. 2012;233(1):62–70. doi:10.1016/j.bbr.2012.04.050.	2
Mainero C, Caramia F, Pozzilli C et al. fMRI evidence of brain reorganization during attention and memory tasks in multiple sclerosis. *NeuroImage*. 2004;21(3):858–867. doi:10.1016/j.neuroimage.2003.10.004.	2
Manelis A, Reder LM. Effective connectivity among the working memory regions during preparation for and during performance of the n-back task. *Frontiers in Human Neuroscience*. 2014;8:593. doi:10.3389/fnhum.2014.00593.	1
Manelis A, Reder LM. He who is well prepared has half won the battle: an FMRI study of task preparation. *Cerebral Cortex*. 2015;25(3):726–735. doi:10.1093/cercor/bht262.	1
Manoach DS, Gollub RL, Benson ES et al. Schizophrenic subjects show aberrant fMRI activation of dorsolateral prefrontal cortex and basal ganglia during working memory performance. *Biological Psychiatry*. 2000;48(2):99–109. doi:10.1016/s0006-3223(00)00227-4.	4
Manoach DS, Greve DN, Lindgren KA, Dale AM. Identifying regional activity associated with temporally separated components of working memory using event-related functional MRI. *NeuroImage*. 2003;20(3):1670–1684. doi:10.1016/j.neuroimage.2003.08.002.	1
Marquand AF, Mourão-Miranda J, Brammer MJ et al. Neuroanatomy of verbal working memory as a diagnostic biomarker for depression. *NeuroReport*. 2008;19(15):1507–1511. doi:10.1097/WNR.0b013e328310425e.	2
Martinkauppi S, Rämä P, Aronen HJ, Korvenoja A, Carlson S. Working memory of auditory localization. *Cerebral Cortex*. 2000;10(9):889–898. doi:10.1093/cercor/10.9.889.	1
Maruishi M, Miyatani M, Nakao T, Muranaka H. Compensatory cortical activation during performance of an attention task by patients with diffuse axonal injury: a functional magnetic resonance imaging study. *Journal of Neurology, Neurosurgery & Psychiatry*. 2007;78(2):168–173. doi:10.1136/jnnp.2006.097345.	2

© 2025 Kutsche J et al. *JAMA Network Open*.

Reference	Table
Marvel CL, Desmond JE. The contributions of cerebro-cerebellar circuitry to executive verbal working memory. *Cortex*. 2010;46(7):880–895. doi:10.1016/j.cortex.2009.08.017.	1
Matsuo K, Glahn DC, Peluso MAM et al. Prefrontal hyperactivation during working memory task in untreated individuals with major depressive disorder. *Molecular Psychiatry*. 2007;12(2):158–166. doi:10.1038/sj.mp.4001894.	2
Mayer JS, Bittner RA, Nikolić D et al. Common neural substrates for visual working memory and attention. *NeuroImage*. 2007;36(2):441–453. doi:10.1016/j.neuroimage.2007.03.007.	1
McAllister TW, Saykin AJ, Flashman LA et al. Brain activation during working memory 1 month after mild traumatic brain injury: a functional MRI study. *Neurology*. 1999;53(6):1300–1308. doi:10.1212/wnl.53.6.1300.	3
McGeown WJ, Shanks MF, Venneri A. Prolonged cholinergic enrichment influences regional cortical activation in early Alzheimer's disease. *Neuropsychiatric Disease and Treatment.* 2008;4(2):465–476. doi:10.2147/ndt.s2461.	2a
McNab F, Leroux G, Strand F et al. Common and unique components of inhibition and working memory: an fMRI, within-subjects investigation. *Neuropsychologia*. 2008;46(11):2668–2682. doi:10.1016/j.neuropsychologia.2008.04.023.	2
Meisenzahl EM, Scheuerecker J, Zipse M et al. Effects of treatment with the atypical neuroleptic quetiapine on working memory function: a functional MRI follow-up investigation. *European Archives of Psychiatry and Clinical Neuroscience.* 2006;256:522–531. doi:10.1007/s00406-006-0687-x.	3
Menon V, Anagnoson RT, Mathalon DH, Glover GH, Pfefferbaum A. Functional neuroanatomy of auditory working memory in schizophrenia: relation to positive and negative symptoms. *NeuroImage*. 2001:13(3):433–446. doi:10.1006/nimg.2000.0699.	Text
Migo EM, Mitterschiffthaler M, O'Daly O et al. Alterations in working memory networks in amnestic mild cognitive impairment. *Aging, Neuropsychology, and Cognition*. 2015;22(1):106–127. doi:10.1080/13825585.2014.894958.	3
Monks PJ, Thompson JM, Bullmore ET et al. A functional MRI study of working memory task in euthymic bipolar disorder: evidence for task-specific dysfunction. *Bipolar Disorders*. 2004;6(6):550–564. doi:10.1111/j.1399-5618.2004.00147.x.	1, 3
Mu Q, Mishory A, Johnson KA et al. Decreased brain activation during a working memory task at rested baseline is associated with vulnerability to sleep deprivation. *Sleep*. 2005;28(4):433–446. doi:10.1093/sleep/28.4.433.	2
Nebel K, Wiese H, Stude P et al. On the neural basis of focused and divided attention. *Cognitive Brain Research*. 2005;25(3):760–776. doi:10.1016/j.cogbrainres.2005.09.011.	3
Nyberg L, Dahlin E, Neely AS, Bäckman L. Neural correlates of variable working memory load across adult age and skill: dissociative patterns within the fronto-parietal network. *Scandinavian Journal of Psychology*. 2009;50(1):41–46. doi:10.1111/j.1467-9450.2008.00678.x.	3
Otsuka Y, Osaka N, Morishita M, Kondo H, Osaka M. Decreased activation of anterior cingulate cortex in the working memory of the elderly. *NeuroReport*. 2006;17(14):1479–1482. doi:10.1097/01.wnr.0000236852.63092.9f.	1
Perlstein WM, Cole MA, Demery JA et al. Parametric manipulation of working memory load in traumatic brain injury: behavioral and neural correlates. *Journal of the International Neuropsychological Society*. 2004;10(5):724–741. doi:10.1017/S1355617704105110.	5
Perlstein WM, Dixit NK, Carter CS, Noll DC, Cohen JD. Prefrontal cortex dysfunction mediates deficits in working memory and prepotent responding in schizophrenia. *Biological Psychiatry*. 2003;53(1):25–38. doi:10.1016/s0006-3223(02)01675-x.	2
Pessoa L, Gutierrez E, Bandettini P, Ungerleider L. Neural correlates of visual working memory: fMRI amplitude predicts task performance. *Neuron*. 2002;35(5):975–987. doi:10.1016/s0896-6273(02)00817-6.	1

Reference	Table
Pfefferbaum A, Desmond JE, Galloway C et al. Reorganization of frontal systems used by alcoholics for spatial working memory: an fMRI study. *NeuroImage*. 2001;14(1):7–20. doi:10.1006/nimg.2001.0785.	2
Picchioni M, Matthiasson P, Broome M et al. Medial temporal lobe activity at recognition increases with the duration of mnemonic delay during an object working memory task. *Human Brain Mapping*. 2007;28(11):1235–1250. doi:10.1002/hbm.20357.	2
Piekema C, Rijpkema M, Fernández G, Kessels RPC. Dissociating the Neural Correlates of Intra-Item and Inter-Item Working-Memory Binding. *PLOS ONE*. 2010;5(4):e10214. doi:10.1371/journal.pone.0010214.	1
Pochon JB, Levy R, Poline JB et al. The role of dorsolateral prefrontal cortex in the preparation of forthcoming actions: an fMRI study. *Cerebral Cortex*. 2001;11(3):260–266. doi:10.1093/cercor/11.3.260.	1
Postle BR, Hamidi M. Nonvisual codes and nonvisual brain areas support visual working memory. *Cerebral Cortex*. 2007;17(9):2151–2162. doi:10.1093/cercor/bhl123.	1
Prakash RS, Heo S, Voss MW, Patterson B, Kramer AF. Age-related differences in cortical recruitment and suppression: Implications for cognitive performance. *Behavioural Brain Research*. 2012;230(1):192–200. doi:10.1016/j.bbr.2012.01.058.	2
Qin S, Hermans EJ, van Marle HJF et al. Acute psychological stress reduces working memory-related activity in the dorsolateral prefrontal cortex. *Biological Psychiatry*. 2009;66(1):25–32. doi:10.1016/j.biopsych.2009.03.006.	1
Quintana J, Wong T, Ortiz-Portillo E et al. Prefrontal-posterior parietal networks in schizophrenia: primary dysfunctions and secondary compensations. *Biological Psychiatry*. 2003;53(1):12–24. doi:10.1016/s0006-3223(02)01435-x.	1
Ragland JD, Gur RC, Valdez J et al. Event-Related fMRI of Frontotemporal Activity During Word Encoding and Recognition in Schizophrenia. *American Journal of Psychiatry*. 2004;161(6):1004–1015. doi:10.1176/appi.ajp.161.6.1004.	2, 3
Ragland JD, Turetsky BI, Gur RC et al. Working Memory for Complex Figures: An fMRI Comparison of Letter and Fractal *n*-Back Tasks. *Neuropsychology*. 2002;16(3):370–379. doi:10.1037/0894-4105.16.3.370.	2
Rämä P, Martinkauppi S, Linnankoski I et al. Working memory of identification of emotional vocal expressions: an fMRI study. *NeuroImage*. 2001;13(6):1090–1101. doi:10.1006/nimg.2001.0777.	1
Ravizza SM, Delgado MR, Chein JM, Becker JT, Fiez JA. Functional dissociations within the inferior parietal cortex in verbal working memory. *NeuroImage*. 2004;22(2):562–573. doi:10.1016/j.neuroimage.2004.01.039.	1
Relander K, Rämä P. Separate neural processes for retrieval of voice identity and word content in working memory. *Brain Research*. 2009;1252:143–151. doi:10.1016/j.brainres.2008.11.050.	1, 2, 3
Rémy F, Mirrashed F, Campbell B, Richter W. Verbal episodic memory impairment in Alzheimer's disease: a combined structural and functional MRI study. *NeuroImage*. 2005;25(1):253–266. doi:10.1016/j.neuroimage.2004.10.045.	3
Reynolds JR, West R, Braver T. Distinct Neural Circuits Support Transient and Sustained Processes in Prospective Memory and Working Memory. *Cerebral Cortex*. 2009;19(5):1208–1221. doi:10.1093/cercor/bhn164.	2
Ricciardi E, Bonino D, Gentili C, Sani L, Pietrini P, Vecchi T. Neural correlates of spatial working memory in humans: a functional magnetic resonance imaging study comparing visual and tactile processes. *Neuroscience*. 2006;139(1):339–349. doi:10.1016/j.neuroscience.2005.08.045.	1
Rodriguez-Jimenez R, Avila C, Garcia-Navarro C et al. Differential dorsolateral prefrontal cortex activation during a verbal n-back task according to sensory modality. *Behavioural Brain Research*. 2009;205:299–302. doi:10.1016/j.bbr.2009.08.022.	1

© 2025 Kutsche J et al. *JAMA Network Open*.

Reference	Table
Sabri M, Humphries C, Verber M et al. Neural effects of cognitive control load on auditory selective attention. *Neuropsychologia*. 2014;61:269–279. doi:10.1016/j.neuropsychologia.2014.06.009.	1
Sánchez-Carrión R, Gómez PV, Junqué C et al. Frontal hypoactivation on functional magnetic resonance imaging in working memory after severe diffuse traumatic brain injury. *Journal of Neurotrauma*. 2008;25(5):479–494. doi:10.1089/neu.2007.0417.	3, 4
Savini N, Brunetti M, Babiloni C, Ferretti A. Working memory of somatosensory stimuli: An fMRI study. *International Journal of Psychophysiology*. 2012;86(3):220–228. doi:10.1016/j.ijpsycho.2012.09.007.	2
Scheuerecker J, Ufer S, Zipse M et al. Cerebral changes and cognitive dysfunctions in medication-free schizophrenia - an fMRI study. *Journal of Psychiatry Research*. 2008;42(6):469–476. doi:10.1016/j.jpsychires.2007.04.001.	2
Schlösser RGM, Koch K, Wagner G et al. Inefficient executive cognitive control in schizophrenia is preceded by altered functional activation during information encoding: an fMRI study. *Neuropsychologia*. 2008;46(1):336–347. doi:10.1016/j.neuropsychologia.2007.07.006.	4b
Schmidt H, Jogia J, Fast K et al. No gender differences in brain activation during the N-back task: an fMRI study in healthy individuals. *Human Brain Mapping*. 2009;30(11):3609–3615. doi:10.1002/hbm.20783.	1
Schneiders JA, Opitz B, Krick CM, Mecklinger A. Separating intra-modal and across-modal training effects in visual working memory: an fMRI investigation. *Cerebral Cortex*. 2011;21(11):2555–2564. doi:10.1093/cercor/bhr037.	1
Schulze K, Gaab N, Schlaug G. Perceiving pitch absolutely: Comparing absolute and relative pitch possessors in a pitch memory task. *BMC Neuroscience*. 2009;10:106. doi:10.1186/1471-2202-10-106.	1
Seo J, Lee BK, Jin SU et al. Lead-Induced Impairments in the Neural Processes Related to Working Memory Function. *PLOS ONE*. 2014;9(8):e105308. doi:10.1371/journal.pone.0105308.	2
Seo J, Kim SH, Kim YT et al. Working memory impairment in fibromyalgia patients associated with altered frontoparietal memory network. *PLOS ONE*. 2012;7(6):e37808. doi:10.1371/journal.pone.0037808.	4
Shen L, Hu X, Yacoub E, Ugurbil K. Neural correlates of visual form and visual spatial processing. *Human Brain Mapping*. 1999;8(1):60–71. doi:10.1002/(SICI)1097-0193(1999)8:1<60::AID-HBM5>3.3.CO;2-Y.	1
Simons JS, Davis SW, Gilbert SJ, Frith CD, Burgess PW. Discriminating imagined from perceived information engages brain areas implicated in schizophrenia. *NeuroImage*. 2006;32(2)_696–703. doi:10.1016/j.neuroimage.2006.04.209.	2
Spreng RN, DuPre E, Selarka D et al. Goal-congruent default network activity facilitates cognitive control. *Journal of Neuroscience*. 2014;34(42):14108–14114. doi:10.1523/JNEUROSCI.2815-14.2014.	1
Stern CE, Owen AM, Tracey I, Look RB, Rosen BR, Petrides M. Activity in ventrolateral and mid-dorsolateral prefrontal cortex during nonspatial visual working memory processing: evidence from functional magnetic resonance imaging. *NeuroImage*. 2000;11(5):392–399. doi:10.1006/nimg.2000.0569.	1
Stoeckel MC, Weder B, Binkofski F et al. A fronto-parietal circuit for tactile object discrimination: an event-related fMRI study. *NeuroImage*. 2003;19(3):1103–1114. doi:10.1016/s1053-8119(03)00182-4.	1, 4
Takeuchi H, Taki Y, Nouchi R et al. Associations among imaging measures (2): the association between gray matter concentration and task-induced activation changes. *Human Brain Mapping*. 2014;35(1):185–198. doi:10.1002/hbm.22167.	3, 5

© 2025 Kutsche J et al. *JAMA Network Open*.

Reference	Table
Thomas RJ, Rosen BR, Stern CE et al. Functional imaging of working memory in obstructive sleep-disordered breathing. *Journal of Applied Physiology*. 2005;98(6):2226–2234. doi:10.1152/japplphysiol.01225.2004.	3
Thomason ME, Race E, Burrows B et al. Development of spatial and verbal working memory capacity in the human brain. *Journal of Cognitive Neuroscience*. 2009;21(2):316–332. doi:10.1162/jocn.2008.21028.	4
Townsend J, Bookheimer SY, Foland-Ross LC, Sugar CA, Altshuler LL. fMRI abnormalities in dorsolateral prefrontal cortex during a working memory task in manic, euthymic and depressed bipolar subjects. *Psychiatry Research*. 2010;182(1):22–29. doi:10.1016/j.pscychresns.2009.11.010.	3
Tregellas JR, Davalos DB, Rojas DC. Effect of task difficulty on the functional anatomy of temporal processing. *NeuroImage*. 2006;32(1):307–315. doi:10.1016/j.neuroimage.2006.02.036.	1
Valera EM, Faraone SV, Biedermann J et al. Functional neuroanatomy of working memory in adults with attention-deficit/hyperactivity disorder. *Biological Psychiatry*. 2005;57(5):439–446. doi:10.1016/j.biopsych.2004.11.034.	3
van den Heuvel OA, Veltman DJ, Groenewegen HJ et al. Frontal-striatal dysfunction during planning in obsessive-compulsive disorder. *Archives of General Psychiatry*. 2005;62(3):301–309. doi:10.1001/archpsyc.62.3.301.	4, 5
Veltman DJ, de Ruiter MB, Rombouts SARB et al. Neurophysiological correlates of increased verbal working memory in high-dissociative participants: a functional MRI study. 2005;35(2):175–185. doi:10.1017/s0033291704002971.	2
Veltman DJ, Rombouts SARB, Dolan RJ. Maintenance versus manipulation in verbal working memory revisited: an fMRI study. *NeuroImage*. 2003;18(2):247–256. doi:10.1016/s1053-8119(02)00049-6.	1, 3
Volle E, Pochon JB, Lehéricy S, Pillon B, Dubois B, Levy R. Specific Cerebral Networks for Maintenance and Response Organization within Working Memory as Evidenced by the 'Double Delay/Double Response' Paradigm. *Cerebral Cortex*. 2005;15(7):1064–1074. doi:10.1093/cercor/bhh207.	1
Walitt B, Čeko M, Khatiwada M et al. Characterizing "fibrofog": Subjective appraisal, objective performance, and task-related brain activity during a working memory task. *NeuroImage: Clinical*. 2016;11:173–180. doi:10.1016/j.nicl.2016.01.021.	2
Walter H, Wolf RC, Spitzer M, Vasic N. Increased left prefrontal activation in patients with unipolar depression: an event-related, parametric, performance-controlled fMRI study. *Journal of Affective Disorders*. 2007;101(1–3):175–185. doi:10.1016/j.jad.2006.11.017.	4
Wildgruber D, Kischka U, Ackermann H, Klose U, Grodd W. Dynamic pattern of brain activation during sequencing of word strings evaluated by fMRI. *Cognitive Brain Research*. 1999;7(3):285–294. doi:10.1016/S0926-6410(98)00031-7.	4
Wishart HA, Saykin AJ, Rabin LA et al. Increased brain activation during working memory in cognitively intact adults with the APOE epsilon4 allele. *American Journal of Psychiatry*. 2006;163(9):1603–1610. doi: 10.1176/ajp.2006.163.9.1603.	2
Yan X, Zhang J, Gong Q, Weng X. Prolonged high-altitude residence impacts verbal working memory: an fMRI study. *Experimental Brain Research*. 2011;208:437–445. doi:10.1007/s00221-010-2494-x.	2
Yi Y, Diesen N, Leung HC. Behavioral and Neural Correlates of Memory Selection and Interference Resolution during a Digit Working Memory Task. *Cognitive, Affective, & Behavioral Neuroscience*. 2009;9:249–259. doi:10.3758/CABN.9.3.249.	1
Yoo SS, Choi BG, Juh RH et al. Working memory processing of facial images in schizophrenia: fMRI investigation. *International Journal of Neuroscience*. 2005;115(3):351–366. doi:10.1080/00207450590520957.	2

© 2025 Kutsche J et al. *JAMA Network Open*.

Reference	Table
Yoo SS, Paralkar G, Panych LP. Neural substrates associated with the concurrent performance of dual working memory tasks. *International Journal of Neuroscience*. 2004;114(6):613–631. doi:10.1080/00207450490430561.	1
Ziemus B, Baumann O, Luerding R et al. Impaired working-memory after cerebellar infarcts paralleled by changes in BOLD signal of a cortico-cerebellar circuit. *Neuropsychologia*. 2007;45(9):2016–2024. doi:10.1016/j.neuropsychologia.2007.02.012.	2

© 2025 Kutsche J et al. *JAMA Network Open*.

eFigure 3.

Replicating the coordinate network mapping approach for these independent sets of coordinates, we find a significant sensitive and specific spot close to and partly overlapping with the right frontopolar hub region of the creativity circuit (outlined in red in the conjunction map)

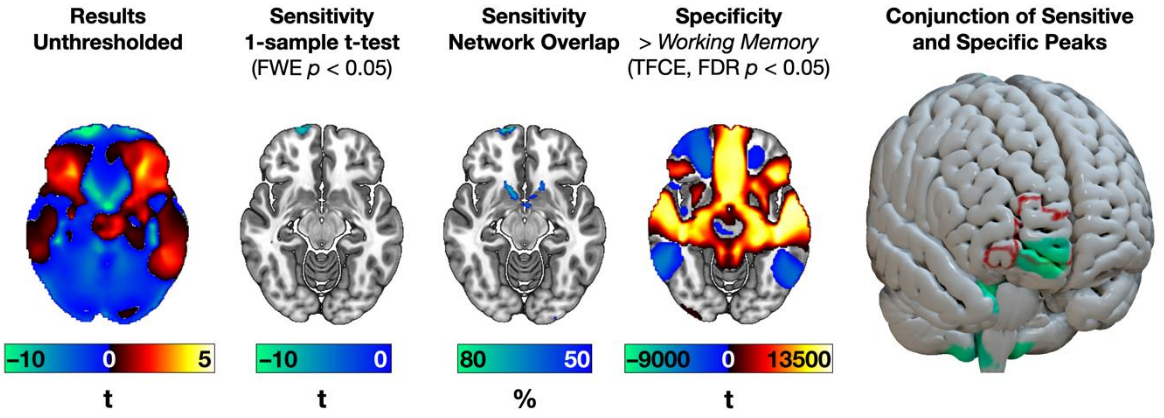

© 2025 Kutsche J et al. *JAMA Network Open*.

eTable 5.

Studies reporting atrophy coordinates for several neurodegenerative diseases including behavioral variant of frontotemporal dementia (bvFTD), semantic (svPPA), logopenic (lvPPA) and non-fluent (nfvPPA) variants of primary progressive aphasia, Parkinson's disease (PD), Alzheimer's disease (AD), and amyotropic lateral sclerosis (ALS)

Disease	Reference	Table
bvFTD	Agosta F, Scola E, Canu E. et al. White Matter Damage in Frontotemporal Lobar Degeneration Spectrum. *Cerebral Cortex*. 2012;22:2705–2714. doi:10.1093/cercor/bhr288.	S4
bvFTD	Ash S, Moore P, Vesely L, et al. Non-fluent speech in frontotemporal lobar degeneration. *Journal of Neurolinguistics*. 2009;22:370–383. doi:10.1016/j.jneuroling.2008.12.001.	4
bvFTD	Baez S, Pinasco C, Roca M, et al. Brain structural correlates of executive and social cognition profiles in behavioral variant frontotemporal dementia and elderly bipolar disorder. *Neuropsychologia*. 2019;126:159–169. doi:10.1016/j.neuropsychologia.2017.02.012.	3
bvFTD	Buhour MS, Doidy F, Laisney M et al. Pathophysiology of the behavioral variant of frontotemporal lobar degeneration: A study combining MRI and FDG-PET. *Brain Imaging and Behavior*. 2017;11:240–252. doi:10.1007/s11682-016-9521-x.	1
bvFTD	Delvecchio G, Mandolini GM, Arighi A, et al. Structural and metabolic cerebral alterations between elderly bipolar disorder and behavioural variant frontotemporal dementia: A combined MRI-PET study. *Australian & New Zealand Journal of Psychiatry*. 2019;53(5):413–423. doi:10.1177/0004867418815976.	2
bvFTD	Dermody N, Wong S, Ahmed R, Piguet O, Hodges JR, Irish M. Uncovering the Neural Bases of Cognitive and Affective Empathy Deficits in Alzheimer's Disease and the Behavioral-Variant of Frontotemporal Dementia. *Journal of Alzheimer's Disease*. 2016;53:801–816. doi:10.3233/JAD-160175.	3
bvFTD	Farb NAS, Grady CL, Strother S, et al. Abnormal network connectivity in frontotemporal dementia: Evidence for prefrontal isolation. *Cortex*. 2013;49:1856–1873. doi:10.1016/j.cortex.2012.09.008.	S2
bvFTD	Filippi M, Agosta F, Scola E, et al. Functional network connectivity in the behavioral variant of frontotemporal dementia. *Cortex*. 2013;49:2389–2401. doi:10.1016/j.cortex.2012.09.017.	e-2
bvFTD	García-Cordero I, Sedeño L, Fraiman D, et al. Stroke and Neurodegeneration Induce Different Connectivity Aberrations in the Insula. *Stroke*. 2015;46:2673–2677. doi: 10.1161/STROKEAHA.115.009598.	S2
bvFTD	Grossman M, McMillan C, Moore P, et al. What's in a name: voxel-based morphometric analyses of MRI and naming difficulty in Alzheimer's disease, frontotemporal dementia and corticobasal degeneration. *Brain*. 2004;127:628–649. doi:10.1093/brain/awh075.	4
bvFTD	Hornberger M, Geng J, Hodges JR. Convergent grey and white matter evidence of orbitofrontal cortex changes related to disinhibition in behavioural variant frontotemporal dementia. *Brain*. 2011;134:2502–2512. doi:10.1093/brain/awr173.	2
bvFTD	Irish M, Piguet O, Hodges JR, Hornberger M. Common and Unique Gray Matter Correlates of Episodic Memory Dysfunction in Frontotemporal Dementia and Alzheimer's Disease. *Human Brain Mapping*. 2014;35:1422–1435. doi:10.1002/hbm.22263.	2
bvFTD	Kipps CM, Nestor PJ, Acosta-Cabronero J, Arnold R, Hodges JR. BRAINA JOURNAL OF NEUROLOGY Understanding social dysfunction in the behavioural variant of frontotemporal dementia: the role of emotion and sarcasm processing. *Brain*. 2009;132:592–603. doi:10.1093/brain/awn314.	2

© 2025 Kutsche J et al. *JAMA Network Open*.

Disease	Reference	Table
bvFTD	Lagarde J, Valabrègue R, Corvol J-C, et al. Why do patients with neurodegenerative frontal syndrome fail to answer: ‘In what way are an orange and a banana alike?’. *Brain*. 2015;138:456–471. doi:10.1093/brain/awu359.	S1
bvFTD	Lee SE, Khazenzon AM, Trujillo AJ, et al. Altered network connectivity in frontotemporal dementia with *C9orf72* hexanucleotide repeat expansion. *Brain*. 2014;137:3047–3060. doi:10.1093/brain/awu248.	S1
bvFTD	Libon DJ, McMillan C, Gunawardena D et al. Neurocognitive contributions to verbal fluency deficits in frontotemporal lobar degeneration. *Neurology*. 2009;73(7):535–542. doi:10.1212/WNL.0b013e3181b2a4f5.	2
bvFTD	Mandelli ML, Vitali P, Santos M, et al. Two insular regions are differentially involved in behavioral variant FTD and nonfluent/agrammatic variant PPA. *Cortex*. 2016;74:149–157. doi:10.1016/j.cortex.2015.10.012.	4
bvFTD	Massimo L, Libon DJ, Chandrasekaran K, et al. Self-appraisal in behavioural variant frontotemporal degeneration. *Journal of Neurology, Neurosurgery and Psychiatry*. 2013;84:148–153. doi:10.1136/jnnp-2012-303153.	E-1
bvFTD	Melloni M, Billeke P, Baez S, et al. Your perspective and my benefit: multiple lesion models of self-other integration strategies during social bargaining. *Brain*. 2016;139(11):1–19. doi:10.1093/brain/aww231.	S1
bvFTD	Pardini M, Huey ED, Cavanagh AL, Grafman J. Olfactory Function in Corticobasal Syndrome and Frontotemporal Dementia. *Archives of Neurology*. 2009;66(1):92–96. doi:10.1001/archneurol.2008.521.	3
bvFTD	Pereira JMS, Williams GB, Acosta-Cabronero J, Pengas G, Spillantini MG, Xuereb JH, Hodges JR, Nestor PJ. Atrophy patterns in histologic vs clinical groupings of frontotemporal lobar degeneration. *Neurology*. 2009;72(19):1653–1660. doi:10.1212/WNL.0b013e3181a55fa2.	Text
bvFTD	Rosen HJ, Gorno-Tempini ML, Goldman WP, Perry RJ, Schuff N, Weiner M, et al. Patterns of brain atrophy in frontotemporal dementia and semantic dementia. *Neurology*. 2002;58:198–208. doi:10.1212/wnl.58.2.198.	4
bvFTD	Seeley WW, Crawford R, Rascovsky K, Kramer JH, Weiner M, Miller BL, Gorno-Tempini ML. Frontal Paralimbic Network Atrophy in Very Mild Behavioral Variant Frontotemporal Dementia. *Archives of Neurology*. 2008;65(2):249–255. doi:10.1001/archneurol.2007.38.	eTable
bvFTD	Yokoyama JS, Bonham LW, Sturm VE, et al. The 5-HTTLPR variant in the serotonin transporter gene modifies degeneration of brain regions important for emotion in behavioral variant frontotemporal dementia: Serotonin transporter in bvFTD. *NeuroImage: Clinical*. 2015;9:283–290. doi:10.1016/j.nicl.2015.07.017.	Text
svPPA	Adlam ALR, Patterson K, Rogers TT, Nestor PJ, Salmond CH, Acosta-Cabronero J, Hodges JR. Semantic dementia and fluent primary progressive aphasia: two sides of the same coin? *Brain*. 2006;129(11):3066–3080. doi:10.1093/brain/awl285.	5
svPPA	Agosta F, Scola E, Canu E. et al. White Matter Damage in Frontotemporal Lobar Degeneration Spectrum. *Cerebral Cortex*. 2012;22:2705–2714. doi:10.1093/cercor/bhr288.	S6
svPPA	Ash S, Moore P, Vesely L, et al. Non-fluent speech in frontotemporal lobar degeneration. *Journal of Neurolinguistics*. 2009;22:370–383. doi:10.1016/j.jneuroling.2008.12.001.	4
svPPA	Ash S, Evans E, O’Shea J, et al. Differentiating primary progressive aphasias in a brief sample of connected speech. *Neurology*. 2013;81(4):329–336. doi:10.1212/WNL.0b013e31829c5d0e.	e-3

© 2025 Kutsche J et al. *JAMA Network Open*.

Disease	Reference	Table
svPPA	Bisenius S, Mueller K, Diehl-Schmid J, et al. Predicting primary progressive aphasias with support vector machine approaches in structural MRI data. *NeuroImage: Clinical*. 2017;14:334–343. doi:10.1016/j.nicl.2017.02.003.	A2
svPPA	Botha H, Duffy JR, Whitwell JL, et al. Classification and clinicoradiologic features of primary progressive aphasia (PPA) and apraxia of speech. *Cortex*. 2015;69:220–236. doi:10.1016/j.cortex.2015.05.013.	C3
svPPA	Boxer AL, Rankin KP, Miller BL, et al. Cinguloparietal atrophy distinguishes Alzheimer disease from semantic dementia. *Archives of Neurology*. 2003;60:949–956. doi:10.1001/archneur.60.7.949.	2
svPPA	Brambati SM, Rankin KP, Narvid J, Seeley WW, Dean D, Rosen HJ, Miller BL, Ashburner J, Gorno-Tempini ML. Atrophy progression in semantic dementia with asymmetric temporal involvement: A tensor-based morphometry study. *Neurobiology of Aging*. 2009;30:103–111. doi:10.1016/j.neurobiolaging.2007.05.014.	2
svPPA	Collins JA, Montal V, Hochberg D, et al. Focal temporal pole atrophy and network degeneration in semantic variant primary progressive aphasia. *Brain*. 2017;140:457–471. doi:10.1093/brain/aww313.	Text
svPPA	Desgranges B, Matuszewski V, Piolino P, et al. Anatomical and functional alterations in semantic dementia: A voxel-based MRI and PET study. *Neurobiology of Aging*. 2007;28:1904–1913. doi:10.1016/j.neurobiolaging.2006.08.006.	2
svPPA	Farb NAS, Grady CL, Strother S, et al. Abnormal network connectivity in frontotemporal dementia: Evidence for prefrontal isolation. *Cortex*. 2013;49:1856–1873. doi:10.1016/j.cortex.2012.09.008.	S2
svPPA	Gorno-Tempini ML, Dronkers NF, Rankin KP, et al. Cognition and Anatomy in Three Variants of Primary Progressive Aphasia. *Annals of Neurology*. 2004;55(3):335–346. doi:10.1002/ana.10825.	4
svPPA	Grossman M, McMillan C, Moore P, et al. What's in a name: voxel-based morphometric analyses of MRI and naming difficulty in Alzheimer's disease, frontotemporal dementia and corticobasal degeneration. *Brain*. 2004;127:628–649. doi:10.1093/brain/awh075.	4
svPPA	Irish M, Addis DR, Hodges JR, Piguet O. Considering the role of semantic memory in episodic future thinking: evidence from semantic dementia. *Brain*. 2012;135:2178–2191. doi:10.1093/brain/aws119.	3
svPPA	Libon DJ, McMillan C, Gunawardena D et al. Neurocognitive contributions to verbal fluency deficits in frontotemporal lobar degeneration. *Neurology*. 2009;73(7):535–542. doi:10.1212/WNL.0b013e3181b2a4f5.	2
svPPA	Miller ZA, Mandelli ML, Rankin KP, et al. Handedness and language learning disability differentially distribute in progressive aphasia variants. *Brain*. 2013;136:3461–3473. doi:10.1093/brain/awt242.	3
svPPA	Pereira JMS, Williams GB, Acosta-Cabronero J, Pengas G, Spillantini MG, Xuereb JH, Hodges JR, Nestor PJ. Atrophy patterns in histologic vs clinical groupings of frontotemporal lobar degeneration. *Neurology*. 2009;72(19):1653–1660. doi:10.1212/WNL.0b013e3181a55fa2.	Text
svPPA	Rosen HJ, Gorno-Tempini ML, Goldman WP, Perry RJ, Schuff N, Weiner M, et al. Patterns of brain atrophy in frontotemporal dementia and semantic dementia. *Neurology*. 2002;58:198–208. doi:10.1212/wnl.58.2.198.	4
svPPA	Wilson SM, Henry ML, Besbris M, et al. Connected speech production in three variants of primary progressive aphasia. *Brain*. 2010;133:2069–2088. doi:10.1093/brain/awq129.	S2

© 2025 Kutsche J et al. *JAMA Network Open*.

Disease	Reference	Table
lvPPA	Agosta F, Scola E, Canu E. et al. White Matter Damage in Frontotemporal Lobar Degeneration Spectrum. *Cerebral Cortex*. 2012;22:2705–2714. doi:10.1093/cercor/bhr288.	S7
lvPPA	Ash S, Evans E, O'Shea J, et al. Differentiating primary progressive aphasias in a brief sample of connected speech. *Neurology*. 2013;81(4):329–336. doi:10.1212/WNL.0b013e31829c5d0e.	e-2
lvPPA	Bisenius S, Mueller K, Diehl-Schmid J, et al. Predicting primary progressive aphasias with support vector machine approaches in structural MRI data. *NeuroImage: Clinical*. 2017;14:334–343. doi:10.1016/j.nicl.2017.02.003.	A2
lvPPA	Bonner MF, Grossman M. Gray Matter Density of Auditory Association Cortex Relates to Knowledge of Sound Concepts in Primary Progressive Aphasia. *The Journal of Neuroscience*. 2012;32(23):7986–7991. doi:10.1523/JNEUROSCI.6241-11.2012.	3
lvPPA	Botha H, Duffy JR, Whitwell JL, et al. Classification and clinicoradiologic features of primary progressive aphasia (PPA) and apraxia of speech. *Cortex*. 2015;69:220–236. doi:10.1016/j.cortex.2015.05.013.	C5
lvPPA	Gorno-Tempini ML, Dronkers NF, Rankin KP, et al. Cognition and Anatomy in Three Variants of Primary Progressive Aphasia. *Annals of Neurology*. 2004;55(3):335–346. doi:10.1002/ana.10825.	4
lvPPA	Gorno-Tempini ML, Brambati SM, Ginex V, et al. The logopenic/phonological variant of primary progressive aphasia. *Neurology*. 2008;71:1227–1234. doi:10.1212/01.wnl.0000320506.79811.da.	3
lvPPA	Hu WT, McMillan C, Libon D, Leight S, FOrman M, Lee VMY, Trojanowski JQ, Grossman M. Multimodal predictors for Alzheimer disease in nonfluent primary progressive aphasia. *Neurology*. 2010;75:595–602. doi:10.1212/WNL.0b013e3181ed9c52.	e-1
lvPPA	Migliaccio R, Agosta F, Rascovsky K, Karydas A, Bonasera S, Rabinovici GD, Miller BL, Gorno-Tempini ML. Clinical syndromes associated with posterior atrophy: Early age at onset AD spectrum. *Neurology*. 2009;73(19):1571–1578. doi:10.1212/WNL.0b013e3181c0d427.	e-3
lvPPA	Miller ZA, Mandelli ML, Rankin KP, et al. Handedness and language learning disability differentially distribute in progressive aphasia variants. *Brain*. 2013;136:3461–3473. doi:10.1093/brain/awt242.	3
lvPPA	Ramanan S, Marstaller L, Hodges JR, Piguet O, Irish M. Understanding the neural basis of episodic amnesia in logopenic progressive aphasia: A multimodal neuroimaging study. *Cortex*. 2020;125:272–287. doi:10.1016/j.cortex.2019.12.026.	S3
lvPPA	Wilson SM, Henry ML, Besbris M, et al. Connected speech production in three variants of primary progressive aphasia. *Brain*. 2010;133:2069–2088. doi:10.1093/brain/awq129.	S2
nfvPPA	Agosta F, Scola E, Canu E. et al. White Matter Damage in Frontotemporal Lobar Degeneration Spectrum. *Cerebral Cortex*. 2012;22:2705–2714. doi:10.1093/cercor/bhr288.	S5
nfvPPA	Ash S, Moore P, Vesely L, et al. Non-fluent speech in frontotemporal lobar degeneration. *Journal of Neurolinguistics*. 2009;22:370–383. doi:10.1016/j.jneuroling.2008.12.001.	4
nfvPPA	Ash S, Evans E, O'Shea J, et al. Differentiating primary progressive aphasias in a brief sample of connected speech. *Neurology*. 2013;81(4):329–336. doi:10.1212/WNL.0b013e31829c5d0e.	e-1

© 2025 Kutsche J et al. *JAMA Network Open*.

Disease	Reference	Table
nfvPPA	Bisenius S, Mueller K, Diehl-Schmid J, et al. Predicting primary progressive aphasias with support vector machine approaches in structural MRI data. *NeuroImage: Clinical*. 2017;14:334–343. doi:10.1016/j.nicl.2017.02.003.	A2
nfvPPA	Botha H, Duffy JR, Whitwell JL, et al. Classification and clinicoradiologic features of primary progressive aphasia (PPA) and apraxia of speech. *Cortex*. 2015;69:220–236. doi:10.1016/j.cortex.2015.05.013.	C2
nfvPPA	Gorno-Tempini ML, Dronkers NF, Rankin KP, et al. Cognition and Anatomy in Three Variants of Primary Progressive Aphasia. *Annals of Neurology*. 2004;55(3):335–346. doi:10.1002/ana.10825.	4
nfvPPA	Grossman M, McMillan C, Moore P, et al. What's in a name: voxel-based morphometric analyses of MRI and naming difficulty in Alzheimer's disease, frontotemporal dementia and corticobasal degeneration. *Brain*. 2004;127:628–649. doi:10.1093/brain/awh075.	4
nfvPPA	Hu WT, McMillan C, Libon D, Leight S, FOrman M, Lee VMY, Trojanowski JQ, Grossman M. Multimodal predictors for Alzheimer disease in nonfluent primary progressive aphasia. *Neurology*. 2010;75:595–602. doi:10.1212/WNL.0b013e3181ed9c52.	e-1
nfvPPA	Libon DJ, McMillan C, Gunawardena D et al. Neurocognitive contributions to verbal fluency deficits in frontotemporal lobar degeneration. *Neurology*. 2009;73(7):535–542. doi:10.1212/WNL.0b013e3181b2a4f5.	2
nfvPPA	Mandelli ML, Vitali P, Santos M, et al. Two insular regions are differentially involved in behavioral variant FTD and nonfluent/agrammatic variant PPA. *Cortex*. 2016;74:149–157. doi:10.1016/j.cortex.2015.10.012.	3
nfvPPA	Miller ZA, Mandelli ML, Rankin KP, et al. Handedness and language learning disability differentially distribute in progressive aphasia variants. *Brain*. 2013;136:3461–3473. doi:10.1093/brain/awt242.	3
nfvPPA	Pereira JMS, Williams GB, Acosta-Cabronero J, Pengas G, Spillantini MG, Xuereb JH, Hodges JR, Nestor PJ. Atrophy patterns in histologic vs clinical groupings of frontotemporal lobar degeneration. *Neurology*. 2009;72(19):1653–1660. doi:10.1212/WNL.0b013e3181a55fa2.	Text
nfvPPA	Wilson SM, Henry ML, Besbris M, et al. Connected speech production in three variants of primary progressive aphasia. *Brain*. 2010;133:2069–2088. doi:10.1093/brain/awq129.	S2
AD	Agosta F, Pievani M, Sala S et al. White matter damage in Alzheimer disease and its relationship to gray matter atrophy. *Radiology*. 2011;258(3):853–863. doi:10.1148/radiol.10101284.	S-2
AD	Baron JC, Chételat G, Desgranges B et al. *In vivo* mapping of gray matter loss with voxel-based morphometry in mild Alzheimer's disease. *NeuroImage*. 2001;14(2):298–309. doi:10.1006/nimg.2001.0848.	1
AD	Boxer AL, Rankin KP, Miller BL, et al. Cinguloparietal atrophy distinguishes Alzheimer disease from semantic dementia. *Archives of Neurology*. 2003;60:949–956. doi:10.1001/archneur.60.7.949.	2
AD	Bozzali M, Giulietti G, Basile B et al. Damage to the Cingulum Contributes toAlzheimer's Disease Pathophysiology byDeafferentation Mechanism. *Human Brain Mapping*. 2012;33(6):1253–1501. doi:10.1002/hbm.21287.	2
AD	Bozzali M, Filippi M, Magnani G et al. The contribution of voxel-based morphometry in staging patients with mild cognitive impairment. *Neurology*. 2006;67(3):453–460. doi:10.1212/01.wnl.0000228243.56665.c2.	3
AD	Brambati SM, Belleville S, Kergoat MJ et al. Single- and Multiple-Domain Amnestic Mild Cognitive Impairment: Two Sides of the Same Coin. *Dementia and Geriatric Cognitive Disorders*. 2010;28(6):541–549. doi:10.1159/000255240.	2

© 2025 Kutsche J et al. *JAMA Network Open*.

Disease	Reference	Table
AD	Brenneis C, Wenning G, Egger KE et al. Basal forebrain atrophy is a distinctive pattern in dementia with Lewy bodies. *NeuroReport*. 2006;15(2):1711–1714. doi:10.1097/01.wnr.0000136736.73895.03.	3
AD	Brys M, Glodzik L, Mosconi L et al. Magnetic Resonance Imaging Improves Cerebrospinal Fluid Biomarkers in the Early Detection of Alzheimer's Disease. *Journal of Alzheimer's Disease*. 2009;16(2):351–362. doi:10.3233/JAD-2009-0968.	2
AD	Canu E, Agosta F, Spinelli EG et al. White matter microstructural damage in Alzheimer's disease at different ages of onset. *Neurobiology of Aging*. 2013;34(10):2331–2340. doi:10.1016/j.neurobiolaging.2013.03.026.	E-1, E-2
AD	Caroli A, Testa C, Geroldi C et al. Cerebral perfusion correlates of conversion to Alzheimer's disease in amnestic mild cognitive impairment. *Journal of Neurology*. 2007;254(12):1698–1707. doi:10.1007/s00415-007-0631-7.	3
AD	Colloby SJ, O'Brien JT, Taylor JP. Patterns of cerebellar volume loss in dementia with Lewy bodies and Alzheimer′s disease: A VBM-DARTEL study. *Psychiatry Research*. 2014;223(3):187–191. doi:10.1016/j.pscychresns.2014.06.006.	2
AD	Dashjamts T, Yoshiura T, Hiwatashi A et al. Simultaneous Arterial Spin Labeling Cerebral Blood Flow and Morphological Assessments for Detection of Alzheimer's Disease. *Academic Radiology*. 2011;18(12):1492–1499. doi:10.1016/j.acra.2011.07.015.	2
AD	Di Paola M, Macaluso E, Carlesimo GA et al. Episodic memory impairment in patients with Alzheimer's disease is correlated with entorhinal cortex atrophy. A voxel-based morphometry study. *Journal of Neurology*. 2007;254:774–781. doi:10.1007/s00415-006-0435-1.	4
AD	Dos Santos V, Thomann P, Wüstenberg T et al. Morphological cerebral correlates of CERAD test performance in mild cognitive impairment and Alzheimer's disease. *Journal of Alzheimers Disease*. 2011;23(3):411–420. doi:10.3233/JAD-2010-100156.	2
AD	Frisch S, Dukart J, Vogt B et al. Dissociating Memory Networks in Early Alzheimer's Disease and Frontotemporal Lobar Degeneration - A Combined Study of Hypometabolism and Atrophy. *PLOS ONE*. 2013;8(2):e55251. doi:10.1371/journal.pone.0055251.	2
AD	Frisoni GB, Testa C, Zorzan A et al. Detection of grey matter loss in mild Alzheimer's disease with voxel based morphometry. *Journal of Neurology, Neurosurgery & Psychiatry*. 2002;73(3):657–664. doi:0.1136/jnnp.73.6.657.	2
AD	Gili T, Cercignani M, Serra L et al. Regional brain atrophy and functional disconnection across Alzheimer's disease evolution. *Journal of Neurology, Neurosurgery & Psychiatry*. 2011;82(1):58–66. doi:10.1136/jnnp.2009.199935.	4
AD	Guo X, Wang Z, Li K et al. Voxel-based assessment of gray and white matter volumes in Alzheimer's disease. *Neuroscience Letters*. 2010;468(2):146–150. doi:10.1016/j.neulet.2009.10.086.	2
AD	Hirata Y, Matsuda H, Nemoto K et al. Voxel-based morphometry to discriminate early Alzheimer's disease from controls. *Neuroscience Letters* 2005;382(3):269–274. doi:10.1016/j.neulet.2005.03.038.	Text
AD	Imabayashi E, Matsuda H, Tabira T. Comparison between brain CT and MRI for voxel-based morphometry of Alzheimer's disease. *Brain and Behavior*. 2013;3(4):487–493. doi:10.1002/brb3.146.	1
AD	Irish M, Piguet O, Hodges JR, Hornberger M. Common and Unique Gray Matter Correlates of Episodic Memory Dysfunction in Frontotemporal Dementia and Alzheimer's Disease. Human Brain Mapping. 2014;35:1422–1435. doi:10.1002/hbm.22263.	2

© 2025 Kutsche J et al. *JAMA Network Open*.

Disease	Reference	Table
AD	Ishii K, Kawachi T, Sasaki H et al. Voxel-Based Morphometric Comparison Between Early- and Late-Onset Mild Alzheimer's Disease and Assessment of Diagnostic Performance of Z Score Images. *American Journal of Neuroradiology*. 2005;26(2):333–340.	2
AD	Kanda T, Ishii K, Uemura T et al. Comparison of grey matter and metabolic reductions in frontotemporal dementia using FDG-PET and voxel-based morphometric MR studies. *European Journal of Nuclear Medicine and Molecular Imaging*. 2008;35:2227–2234. doi:10.1007/s00259-008-0871-5.	2
AD	Koenig P, Smith EE, Troiani V et al. Medial Temporal Lobe Involvement in an Implicit Memory Task: Evidence of Collaborating Implicit and Explicit Memory Systems from fMRI and Alzheimer's Disease. *Cerebral Cortex*. 2008;18(12):2831–2843. doi:10.1093/cercor/bhn043.	2
AD	Matsunari I, Samuraki M, Chen WP et al. Comparison of 18F-FDG PET and optimized voxel-based morphometry for detection of Alzheimer's disease: aging effect on diagnostic performance. *Journal of Nuclear Medicine*. 2007;48(12):1961–1970. doi:10.2967/jnumed.107.042820.	2
AD	Mazère J, Prunier C, Barret O et al. *In vivo* SPECT imaging of vesicular acetylcholine transporter using [(123)I]-IBVM in early Alzheimer's disease. *NeuroImage*. 2008;40(1):280–288. doi:10.1016/j.neuroimage.2007.11.028.	2
AD	Mok GSP, Wu YY, Lu KM et al. Evaluation of the screening power of Cognitive Abilities Screening Instrument for probable Alzheimer's disease using voxel-based morphometry. *Clinical Imaging.* 2012;36:46–53. doi:10.1016/j.clinimag.2011.03.004.	2
AD	Rabinovici GD, Seeley WW, Kim EJ et al. Distinct MRI atrophy patterns in autopsy-proven Alzheimer's disease and frontotemporal lobar degeneration. *American Journal of Alzheimer's Disease & Other Dementias*. 2007;22(6):474–488. doi:10.1177/1533317507308779.	3
AD	Raji CA, Lopez OL, Kuller LH et al. Age, Alzheimer disease, and brain structure. *Neurology*. 2009;73(22):1899–1905. doi:10.1212/WNL.0b013e3181c3f293.	e-2
AD	Shiino A, Watanabe T, Kitagawa T et al. Different Atrophic Patterns in Early- and Late-Onset Alzheimer's Disease and Evaluation of Clinical Utility of a Method of Regional z-Score Analysis Using Voxel-Based Morphometry. *Dementia and Geriatric Cognitive Disorders*. 2008;26:175–186. doi:10.1159/000151241.	2
AD	Shiino A, Watanabe T, Maeda K et al. Four subgroups of Alzheimer's disease based on patterns of atrophy using VBM and a unique pattern for early onset disease. *NeuroImage*. 2006;33:17–26. doi:10.1016/j.neuroimage.2006.06.010.	2
AD	Takahashi R, Ishii K, Miyamoto N et al. Measurement of gray and white matter atrophy in dementia with Lewy bodies using diffeomorphic anatomic registration through exponentiated lie algebra: A comparison with conventional voxel-based morphometry. *American Journal of Neuroradiology*. 2010;31(10):1873–1878. doi:10.3174/ajnr.A2200.	3
AD	Testa C, Laakso MP, Sabattoli F et al. A comparison between the accuracy of voxel-based morphometry and hippocampal volumetry in Alzheimer's disease. *Journal of Magnetic Resonance Imaging*. 2004;19(3):274–282. doi:10.1002/jmri.20001.	2
AD	Tondelli M, Wilcock GK, Nichelli P et al. Structural MRI changes detectable up to ten years before clinical Alzheimer's disease. *Neurobiology of Aging*. 2012;33:825.e25–825.e36. doi:10.1016/j.neurobiolaging.2011.05.018.	2
AD	Whitwell JL, Jack Jr CR, Przybelski SA et al. Temporoparietal atrophy: A marker of AD pathology independent of clinical diagnosis. *Neurobiology of Aging*. 2011;32(9):1521–1541. doi:10.1016/j.neurobiolaging.2009.10.012.	3

© 2025 Kutsche J et al. *JAMA Network Open*.

Disease	Reference	Table
AD	Xie S, Xiao JX, Gong GL et al. Voxel-based detection of white matter abnormalities in mild Alzheimer disease. *Neurology*. 2006;66(12):1845–1849. doi:10.1212/01.wnl.0000219625.77625.aa.	1
AD	Zahn R, Buechert M, Overmans J et al. Mapping of temporal and parietal cortex in progressive nonfluent aphasia and Alzheimer's disease using chemical shift imaging, voxel-based morphometry and positron emission tomography. *Psychiatry Research*. 2005;140(29):115–131. doi:10.1016/j.pscychresns.2005.08.001.	2
PD	Abe Y, Kachi T, Arahata Y et al. Occipital hypoperfusion in Parkinson's disease without dementia: correlation to impaired cortical visual processing. *Journal of Neurology, Neurosurgery and Psychiatry*. 2003;74:419–422. doi:10.1136/jnnp.74.4.419.	2
PD	Alzahrani H, Antonini A, Vennery A. Apathy in Mild Parkinson's Disease: Neuropsychological and Neuroimaging Evidence. *Journal of Parkinson's Disease*. 2016;6(4):821–832. doi:10.3233/JPD-160809.	3
PD	Berding G, Odin P, Brooks DJ et al. Resting Regional Cerebral Glucose Metabolism in AdvancedParkinson's Disease Studied in the *Off* and *On* Conditions with [^{18}F]FDG-PET. *Movement Disorders*. 2001;16(6):1014–1022. doi:10.1002/mds.1212.	3
PD	Berti V, Polito C, Borghammer P et al. Alternative normalization methods demonstrate widespread cortical hypometabolism in untreated de novo Parkinson's disease. *The Quarterly Journal of Nuclear Medicine and Molecular Imaging*. 2012;56(3):299–308.	2
PD	Burton EJ, McKeith IG, Burn DJ, Williams ED, O'Brien JT. Cerebral atrophy in Parkinson's disease with and without dementia: a comparison with Alzheimer's disease, dementia with Lewy bodies and controls. *Brain*. 2004;127:791–800. doi:10.1093/brain/awh088.	4
PD	Camicioli R, Gee M, Bouchard TP, et al. Voxel-based morphometry reveals extra-nigral atrophy patterns associated with dopamine refractory cognitive and motor impairment in parkinsonism. *Parkinsonism and Related Disorders*. 2009;15:187–195. doi:10.1016/j.parkreldis.2008.05.002.	2
PD	Chen B, Wang S, Sun W, et al. Functional and structural changes in gray matter of parkinson's disease patients with mild cognitive impairment. *European Journal of Radiology*. 2017;93:16–23. doi:10.1016/j.ejrad.2017.05.018.	2
PD	Chung EJ, Han YH, Mun CW et al. Hypometabolism based on a cutoff point on the mini-mental state examination in Parkinson's disease. *Neurology Asia*. 2016;21(3):247–253.	2
PD	Cilia R, Siri C, Marotta G, et al. Functional Abnormalities Underlying Pathological Gambling in Parkinson Disease. *Archives of Neurology*. 2008;65(12):1604–1611. doi:10.1001/archneur.65.12.1604.	3
PD	Compta Y, Ibarretxe-Bilbao N, Perira JB et al. Grey matter volume correlates of cerebrospinal markers of Alzheimer-pathology in Parkinson's disease and related dementia. *Parkinsonism and Related Disorders*. 2012;18(8):941–947. doi:10.1016/j.parkreldis.2012.04.028.	3
PD	Cordato NJ, Duggins AJ, Halliday GM, Morris JGL, Pantelis C. Clinical deficits correlate with regional cerebral atrophy in progressive supranuclear palsy. *Brain*. 2005;128(6):1259–1266. doi:10.1093/brain/awh508.	2
PD	Díez-Cirarda M, Ojeda N, Peña J et al. Neuroanatomical Correlates of Theory of Mind Deficit in Parkinson's Disease: A Multimodal Imaging Study. *PLOS ONE*. 2015;10(11):e0142234. doi:10.1371/journal.pone.0142234.	2

© 2025 Kutsche J et al. *JAMA Network Open*.

Disease	Reference	Table
PD	Fioravanti V, Benuzzi F, Codeluppi L, et al. MRI Correlates of Parkinson's Disease Progression: A Voxel Based Morphometry Study. *Parkinson's Disease*. 2015;2015378032. doi:10.1155/2015/378032.	3
PD	Gama RL, Bruin VMS, Távora DGF, Duran FLS, Bittencourt L, Tufik S. Structural brain abnormalities in patients with Parkinson's disease with visual hallucinations: A comparative voxel-based analysis. *Brain and Cognition*. 2014;87:97–103. doi:10.1016/j.bandc.2014.03.011.	2
PD	Gao Y, Nie K, Huang B, et al. Changes of brain structure in Parkinson's disease patients with mild cognitive impairment analyzed via VBM technology. *Neuroscience Letters*. 2017;658:121–132. doi:10.1016/j.neulet.2017.08.028.	2
PD	Gerrits NJHM, van der Werf YD, Hofman M et al. Gray matter differences contribute to variation in cognitiveperformance in Parkinson's disease. *European Journal of Neurology*. 2014;21:245–252. doi:10.1111/ene.12269.	2
PD	Guimarães RP, Santos MCA, Dagher A, et al. Pattern of reduced Functional connectivity and structural abnormalities in Parkinson's Disease: an exploratory study. *Frontiers in Neurology*. 2017;7243. doi:10.3389/fneur.2016.00243.	2
PD	Hosey L, Thompson J, Metman L et al. Temporal Dynamics of Cortical and Subcortical Responses to Apomorphine in Parkinson Disease: An $H_2^{15}O$ PET Study. *Clinical Neuropharmacology*. 2005;28(1):18–27. doi:10.1097/01.wnf.0000154220.30263.0e.	2
PD	Hosokai Y, Nishio Y, Hirayama K et al. Distinct Patterns of Regional Cerebral Glucose Metabolism in Parkinson's Disease with and without Mild Cognitive Impairment. *Movement Disorders*. 2009;24(6):854–862. doi:10.1002/mds.22444.	3
PD	Hsu JL, Jung TP, Hsu CY et al. Regional CBF changes in Parkinson's disease: A correlation with motor dysfunction. *European Journal of Nuclear Medicine and Molecular Imaging*. 2007;34(9):1458–1466. doi:10.1007/s00259-006-0360-7.	3
PD	Huang C, Ravdin LD, Nirenberg MJ, et al. Neuroimaging Markers of Motor and Nonmotor Features of Parkinson's Disease: An [18F]Fluorodeoxyglucose Positron Emission Computed Tomography Study. *Dementia and Geriatric Cognitive Disorders*. 2013;35:183–196. doi:10.1159/000345987.	2
PD	Huang P, Lou Y, Yuan M et al. Cortical abnormalities in Parkinson's disease patients and relationship to depression: A surface-based morphometry study. *Psychiatry Research: Neuroimaging*. 2016;250:24–28. doi:10.1016/j.pscychresns.2016.03.002.	2
PD	Ilmon Y, Matsuda H, Ogawa M et al. SPECT image analysis using statistical parametric mapping in patients with Parkinson's disease. *Journal of Nuclear Medicine*. 1999;40(10):1583–1589.	3
PD	Jia X, Wang Z, Yang T et al.Entorhinal Cortex Atrophy in Early, Drug-naive Parkinson's Disease with Mild Cognitive Impairment. *Aging and Disease*. 2019;10(6):1221–1232. doi:10.14336/AD.2018.1116.	3
PD	Juh R, Kim J, Moon D, Choe B, Suh T. Different metabolic patterns analysis of Parkinsonism on the ^{18}F-FDG PET. *European Journal of Radiology*. 2004;51:223–233. doi:10.1016/S0720-048X(03)00214-6.	4
PD	Juh R, Pae CU, Lee CU et al. Voxel based comparison of glucose metabolism in the differential diagnosis of the multiple system atrophy using statistical parametric mapping. *Neuroscience Research*. 2005;52:211–219. doi:10.1016/j.neures.2005.03.010.	3
PD	Kikuchi A, Takeda A, Kimpara T et al. Hypoperfusion in the supplementary motor area, dorsolateral prefrontal cortex and insular cortex in Parkinson's disease. *Journal of the Neurological Sciences*. 2001;193(1):29–36. doi:10.1016/S0022-510X(01)00641-4.	2

© 2025 Kutsche J et al. *JAMA Network Open*.

Disease	Reference	Table
PD	Kostić VS, Agosta F, Petrović I et al. Regional patterns of brain tissue loss associated with depression in Parkinson disease. *Neurology*. 2010;75(10):857–863. doi:10.1212/WNL.0b013e3181f11c1d.	3
PD	Le Jeune F, Péron J, Grandjean D et al. Subthalamic nucleus stimulation affects limbic and associative circuits: A PET study. *European Journal of Nuclear Medicine and Molecular Imaging*. 2010;37(8);1512–1520. doi:10.1007/s00259-010-1436-y.	3
PD	Lee EY, Sen S, Eslinger PJ et al. Side of Motor Onset is Associated with Hemisphere-Specific Memory Decline and Lateralized Gray Matter Loss in Parkinson's disease. *Physiology & Behavior*. 2017;176(3):139–148. doi:10.1016/j.parkreldis.2015.02.008.	3
PD	Li X, Xing Y, Schwarz ST, Auer DP. Limbic Grey Matter Changes in Early Parkinson's Disease. *Human Brain Mapping*. 2017;38:3566–3578. doi:10.1002/hbm.23610.	2
PD	Lin C-H, Chen C-M, Lu M-K, et al. VBM reveals brain volume differences between Parkinson's disease and essential tremor patients. *Frontiers in Human Neuroscience*. 2013;7247. doi:10.3389/fnhum.2013.00247.	S2-a
PD	Lyoo CH, Ryu YH, Lee MS. Topographical Distribution of Cerebral Cortical Thinning in Patients with Mild Parkinson's Disease Without Dementia. *Movement Disorders*. 2010;25(4):496–499. doi:10.1002/mds.22975.	1
PD	Martin WRW, Wieler M, Gee M, Camicioli R. Temporal Lobe Changes in Early, Untreated Parkinson's Disease. *Movement Disorders*. 2009;24(13):1949–1954. doi:10.1002/mds.22680.	3
PD	Naduthota RM, Bharath RD, Jhunjhunwala K et al. Imaging biomarker correlates with oxidative stress in Parkinson's disease. *Neurology India*. 2017;65(2):263–268. doi:10.4103/neuroindia.NI_981_15.	2
PD	Nagano-Saito A, Washimi Y, Arahata Y et al. Cerebral atrophy and its relation to cognitive impairment in Parkinson disease. *Neurology*. 2005;64(2):224–229. doi:10.1212/01.WNL.0000149510.41793.50	2
PD	Pagonabarraga J, Soriano-Mas C, Llebaria G et al. Neural correlates of minor hallucinations in non-demented patients with Parkinson's disease. *Parkinsonism and Related Disorders*. 2014;20(3):290–296. doi:10.1016/j.parkreldis.2013.11.017.	S-1
PD	Pagonabarraga J, Corcuera-Solano I, Vives-Gilabert Y et al. Pattern of Regional Cortical Thinning Associated with Cognitive Deterioration in Parkinson's Disease. *PLOS ONE*. 2013;8(1):e54980. doi:10.1371/journal.pone.0054980.	2
PD	Pereira JB, Ibarretxe-Bilbao N, Marti MJ et al. Assessment of cortical degeneration in patients with Parkinson's disease by voxel-based morphometry, cortical folding, and cortical thickness. *Human Brain Mapping*. 2012;33(11):2521–2534. doi:10.1002/hbm.21378.	2
PD	Potgieser ARE, van der Hoorn A, Meppelink AM, Teune LK, Koerts J, de Jong BM. Anterior Temporal Atrophy and Posterior Progression in Patients with Parkinson's Disease. *Neurodegenerative Diseases*. 2014;14:125–132. doi:10.1159/000363245.	2
PD	Ramírez-Ruiz B, Martí MJ, Tolosa E et al. Cerebral atrophy in Parkinson's disease patients with visual hallucinations. *European Journal of Neurology*. 2007;14:750–756. doi:10.1111/j.1468-1331.2007.01768.x.	2
PD	Summerfield C, Junqué C, Tolosa E et al. Structural Brain Changes in Parkinson Disease With Dementia. *Archives of Neurology*. 2005;62:281–285. doi:10.1001/archneur.62.2.281.	2

© 2025 Kutsche J et al. *JAMA Network Open*.

Disease	Reference	Table
PD	Terada T, Miyata J, Obi T, Kubota M, Yoshizumi M, Murai T. Reduced gray matter volume is correlated with frontal cognitive and behavioral impairments in Parkinson's disease. *Journal of the Neurological Sciences*. 2018;390:231–238. doi:10.1016/j.jns.2018.05.005.	2A
PD	Tessitore A, Amboni M, Cirillo G et al. Regional gray matter atrophy in patients with Parkinson disease and freezing of gait. *American Journal of Neuroradiology*. 2012;33(9):1804–1809. doi:10.3174/ajnr.A3066.	2
PD	Tessitore A, Santangelo G, De Micco R et al. Cortical thickness changes in patients with Parkinson's disease and impulse control disorders. *Parkinsonism and Related Disorders*. 2016;24:119–125. doi:10.1016/j.parkreldis.2015.10.013.	3
PD	Teune LK, Bartels AN, de Jong BM et al. Typical Cerebral Metabolic Patterns in Neurodegenerative Brain Diseases. *Movement Disorders*. 2010;25(14):2395–2404. doi:10.1002/mds.23291.	1
PD	Tir M, Delmaire C, le Thuc V et al. Motor-Related Circuit Dysfunction in MSA-P: Usefulness of Combined Whole-Brain Imaging Analysis. *Movement Disorders*. 2009;24(6):793–948. doi:10.1002/mds.22463.	2
PD	Van Laere K, Santens P, Bosman T, et al. Statistical parametric mapping of ^{99m}Tc-ECD SPECT in idiopathic Parkinson’s disease and multiple system atrophy with predominant parkinsonian features: Correlation with clinical parameters. *Journal of Nuclear Medicine.* 2004;45(6):933–942.	2
PD	Wang X, Zhang J, Yuan Y et al. Cerebral metabolic change in Parkinson’s disease patients with anxiety: A FDG-PET study. *Neuroscience Letters*. 2017;653:202–207. doi:10.1016/j.neulet.2017.05.062.	2
PD	Wilson H, Niccolini F, Pellicano C, Politis M. Cortical thinning across Parkinson's disease stages and clinical correlates. *Journal of the Neurological Sciences*. 2019;398:31–38. doi:10.1016/j.jns.2019.01.020.	2
PD	Xuan M, Guan X, Huang P et al. Different patterns of gray matter density in early- and middle-late-onset Parkinson’s disease: a voxel-based morphometry study. *Brain Imaging and Behavior*. 2019;13(1):172–179. doi:10.1007/s11682-017-9745-4.	2
PD	Yadav SK, Kathiresan N, Mohan S et al. Gender-based analysis of cortical thickness and structural connectivity in Parkinson’s disease. *Journal of Neurology*. 2016;263:2308–2318. doi:10.1007/s00415-016-8265-2.	2
PD	Zhang J, Zhang YT, Hu WD et al. Gray matter atrophy in patients with Parkinson’s disease and those with mild cognitive impairment: a voxel-based morphometry study. *International Journal of Clinical and Experimental Medicine*. 2015;8(9):15383–15392.	3
ALS	Abrahams S, Goldstein LH, Suckling J et al. Frontotemporal white matter changes in amyotrophic lateral sclerosis. *Journal of Neurology*. 2005;252:321–331. doi:10.1007/s00415-005-0646-x.	3
ALS	Agosta F, Pagani E, Rocca MA et al. Voxel-based morphometry study of brain volumetry and diffusivity in amyotrophic lateral sclerosis patients with mild disability. *Human Brain Mapping*. 2007;28(12):1430–1438. doi:10.1002/hbm.20364.	1
ALS	Agosta F, Gorno-Tempini ML, Pagani E et al. Longitudinal assessment of grey matter contraction in amyotrophic lateral sclerosis: A tensor based morphometry study. *Amyotrophic Lateral Sclerosis*. 2009;10(3):168–174. doi:10.1080/17482960802603841.	2
ALS	Bede P, Bokde A, Elamin M et al. Grey matter correlates of clinical variables in amyotrophic lateral sclerosis (ALS): a neuroimaging study of ALS motor	Fig. 1, Fig. 5

© 2025 Kutsche J et al. *JAMA Network Open*.

Disease	Reference	Table
	phenotype heterogeneity and cortical focality. *Journal of Neurology, Neurosurgery & Psychiatry*. 2013;84(7):766–773. doi:10.1136/jnnp-2012-302674.	
ALS	Buhour MS, Doidy F, Mondou A et al. Voxel-based mapping of grey matter volume and glucose metabolism profiles in amyotrophic lateral sclerosis. *EJNMMI Research*. 2017;7:21. doi:10.1186/s13550-017-0267-2.	2
ALS	Canu E, Agosta F, Riva N. et al. The Topography of Brain Microstructural Damage in Amyotrophic Lateral Sclerosis Assessed Using Diffusion Tensor MR Imaging. *American Journal of Neuroradiology*. 2011;32(7):1307–1314. doi:10.3174/ajnr.A2469.	2
ALS	Cerami C, Dodich A, Canessa N et al. Emotional empathy in amyotrophic lateral sclerosis: a behavioural and voxel-based morphometry study. *Amyotrophic Lateral Sclerosis and Frontotemporal Degeneration*. 2014;15(1–2):21–29. doi:10.3109/21678421.2013.785568.	3
ALS	Chang JL, Lomen-Hoerth C, Murphy J et al. A voxel-based morphometry study of patterns of brain atrophy in ALS and ALS/FTLD. *Neurology*. 2005;65(1):75–80. doi:10.1212/01.wnl.0000167602.38643.29.	2
ALS	Chen Z, Liu M, Ma L. Gray Matter Volume Changes over the Whole Brain in the Bulbar- and Spinal-onset Amyotrophic Lateral Sclerosis: a Voxel-based Morphometry Study. *Chinese Medical Sciences Journal*. 2018;33(1):20–28. doi:10.24920/11804.	3
ALS	Christidi F, Karavasilis E, Riederer F et al. Gray matter and white matter changes in non-demented amyotrophic lateral sclerosis patients with or without cognitive impairment: A combined voxel-based morphometry and tract-based spatial statistics whole-brain analysis. *Brain Imaging and Behavior*. 2018;12:547–563. doi:10.1007/s11682-017-9722-y.	4
ALS	Cosottini M, Pesaresi I, Piazza S et al. Structural and functional evaluation of cortical motor areas in Amyotrophic Lateral Sclerosis. *Experimental Neurology*. 2012;234(1):169–180. doi:10.1016/j.expneurol.2011.12.024.	S-A
ALS	Cosottini M, Cecchi P, Piazza S et al. Mapping cortical degeneration in ALS with magnetization transfer ratio and voxel-based morphometry. *PLOS ONE*. 2013;8(7):e68279. doi:10.1371/journal.pone.0068279.	S-2
ALS	D'Ambrosio A, Gallo A, Trojsi F et al. Frontotemporal Cortical Thinning in Amyotrophic Lateral Sclerosis. *American Journal of Neuroradiology*. 2014;35(2):304–310. doi:10.3174/ajnr.A3753.	2
ALS	Devine MS, Pannek K, Coulthard A et al. Exposing asymmetric gray matter vulnerability in amyotrophic lateral sclerosis. *NeuroImage Clinical*. 2015;7:782–787. doi:10.1016/j.nicl.2015.03.006.	2
ALS	Ellis CM, Suckling J, Amaro Jr E et al. Volumetric analysis reveals corticospinal tract degeneration and extramotor involvement in ALS. *Neurology*. 2001;57(9):1571–1578. doi:10.1212/wnl.57.9.1571.	3
ALS	Grosskreutz J, Kaufmann J, Frädrich J et al. Widespread sensorimotor and frontal cortical atrophy in Amyotrophic Lateral Sclerosis. *BMC Neurology*. 2006;6:17. doi:10.1186/1471-2377-6-17.	3
ALS	Grossman M, Anderson C, Khan A et al. Impaired action knowledge in amyotrophic lateral sclerosis. *Neurology*. 2008;71(18):1396–1401. doi:10.1212/01.wnl.0000319701.50168.8c.	2
ALS	Kwan JY, Meoded A, Danielian LE et al. Structural imaging differences and longitudinal changes in primary lateral sclerosis and amyotrophic lateral sclerosis. *NeuroImage Clinical*. 2012;2:151–160. doi:10.1016/j.nicl.2012.12.003.	2

Disease	Reference	Table
ALS	Luo CY, Chen Q, Huang R et al. Patterns of Spontaneous Brain Activity in Amyotrophic Lateral Sclerosis: A Resting-State fMRI Study. *PLOS ONE*. 2012;7(9):e45470. doi:10.1371/journal.pone.0045470.	2
ALS	Menke RAL, Körner S, Filippini N et al. Widespread grey matter pathology dominates the longitudinal cerebral MRI and clinical landscape of amyotrophic lateral sclerosis. *Brain*. 2014;137(Pt 9):2546–2555. doi:10.1093/brain/awu162.	Fig. 1
ALS	Meoded A, Kwan JY, Peters TL et al. Imaging findings associated with cognitive performance in primary lateral sclerosis and amyotrophic lateral sclerosis. *Dementia and Geriatric Cognitive Disorders Extra*. 2013;3(1):233–250. doi:10.1159/000353456.	Text
ALS	Mezzapesa DM, Ceccarelli A, Dicuonzo F et al. Whole-Brain and Regional Brain Atrophy in Amyotrophic Lateral Sclerosis. *American Journal of Neuroradiology*. 2007;28(2):255–259.	3
ALS	Minnerop M, Specht K, Ruhlmann J et al. In vivo voxel-based relaxometry in amyotrophic lateral sclerosis. *Journal of Neurology*. 2009;256:28–34. doi:10.1007/s00415-009-0947-6.	2
ALS	Raaphorst J, van Tol MJ, de Visser M et al. Prose memory impairment in amyotrophic lateral sclerosis patients is related to hippocampus volume. *European Journal of Neurology*. 2015;22(3):547–554. doi:10.1111/ene.12615.	A-3
ALS	Sage CA, Peeters RP, Görner A et al. Quantitative diffusion tensor imaging in amyotrophic lateral sclerosis. *NeuroImage*. 2007;34(2):486–499. doi:10.1016/j.neuroimage.2006.09.025.	3
ALS	Senda J, Kato S, Kaga T. et al. Progressive and widespread brain damage in ALS: MRI voxel-based morphometry and diffusion tensor imaging study. *Amyotrophic Lateral Sclerosis*. 2011;12(1):59–69. doi:10.3109/17482968.2010.517850.	S-2
ALS	Stoppel CM, Vielhaber S, Eckart C et al. Structural and functional hallmarks of amyotrophic lateral sclerosis progression in motor- and memory-related brain regions. *NeuroImage Clinical*. 2014;5:277–290. doi:10.1016/j.nicl.2014.07.007.	6
ALS	Tavazzi E, Laganà MM, Bergsland N et al. Grey matter damage in progressive multiple sclerosis versus amyotrophic lateral sclerosis: a voxel-based morphometry MRI study. *Neurological Sciences*. 2015;36:371–377. doi:10.1007/s10072-014-1954-7.	2
ALS	Tedeschi G, Trojsi F, Tessitore A et al. Interaction between aging and neurodegeneration in amyotrophic lateral sclerosis. *Neurobiology of Aging*. 2012;33(5):886–898. doi:10.1016/j.neurobiolaging.2010.07.011.	2
ALS	Thivard L, Pradat PF, Lehéricy S et al. Diffusion tensor imaging and voxel based morphometry study in amyotrophic lateral sclerosis: relationships with motor disability. *Journal of Neurology, Neurosurgery & Psychiatry*. 2007;78:889–892. doi:10.1136/jnnp.2006.101758.	1
ALS	Zhang Q, Mao C, Jin J et al. Side of Limb-Onset Predicts Laterality of Gray Matter Loss in Amyotrophic Lateral Sclerosis. *BioMed Research International*. 2014:473250. doi:10.1155/2014/473250.	5

© 2025 Kutsche J et al. *JAMA Network Open*.

eFigure 4.
Lesions affecting creativity task performance and the creativity circuit

(A) Lesion overlap maps from Abraham et al. (2012) in different areas (top) and their topographical relation to the creativity network (bottom). (B) ANOVA of average ROI-to-ROI correlations between each lesion overlap mask and the individual (n = 36) creativity study-level seeds (in bar graph: * p < 0.01 for a 1-sample t-test; above bar graphs: p < 0.001 for ANOVA). The color bar in (B) indicates the lesion group's average connectivity to the right frontal pole. Positive connectivity to the right frontal pole aligns with a positive correlation with creativity and vice versa.

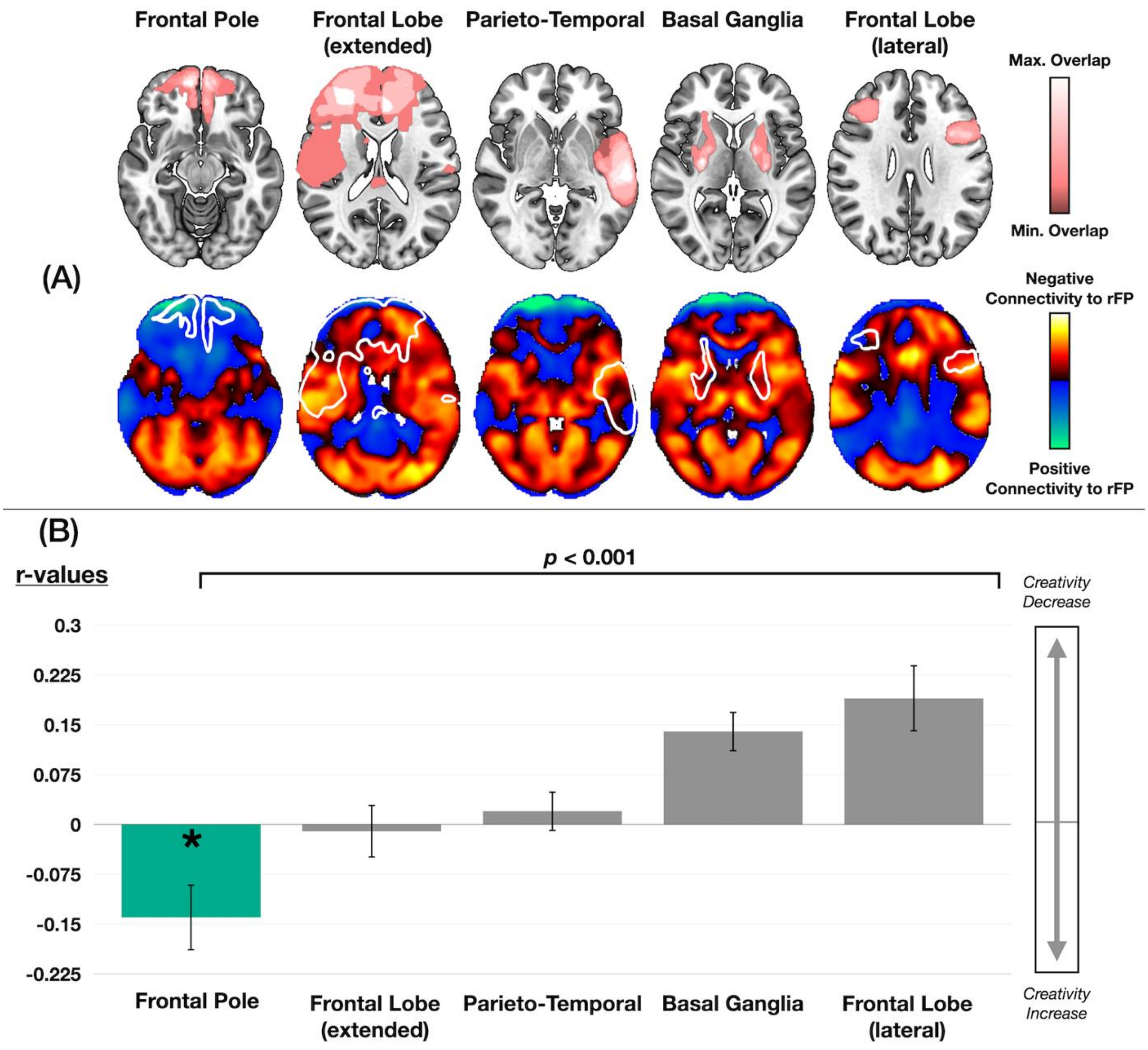

© 2025 Kutsche J et al. *JAMA Network Open*.

eFigure 5.
Neurodegenerative atrophy patterns and the creativity circuit

(A) Atrophy coordinates for semantic (svPPA), logopenic (lvPPA) and nonfluent (nfvPPA) variants of primary progressive aphasia, behavioral variant of frontotemporal dementia (bvFTD), typical Alzheimer's disease (AD), Parkinson's disease (PD) and amyotrophic lateral sclerosis (ALS) in different areas and their topographical relation to the creativity network. For svPPA, 75% of the atrophy coordinates hit regions positively connected to the right frontal pole (rFP) (cool colors). By contrast, for nfvPPA, 68% of the atrophy coordinates hit regions negatively connected to the rFP (warm colors)(B) ANOVA of average ROI-to-ROI correlations between each study-level atrophy seed and the individual (n = 36) creativity study-level seeds (in bar graph: * p < 0.05 for a 1-sample t-test; above bar graphs: p < 0.001 for ANOVA).

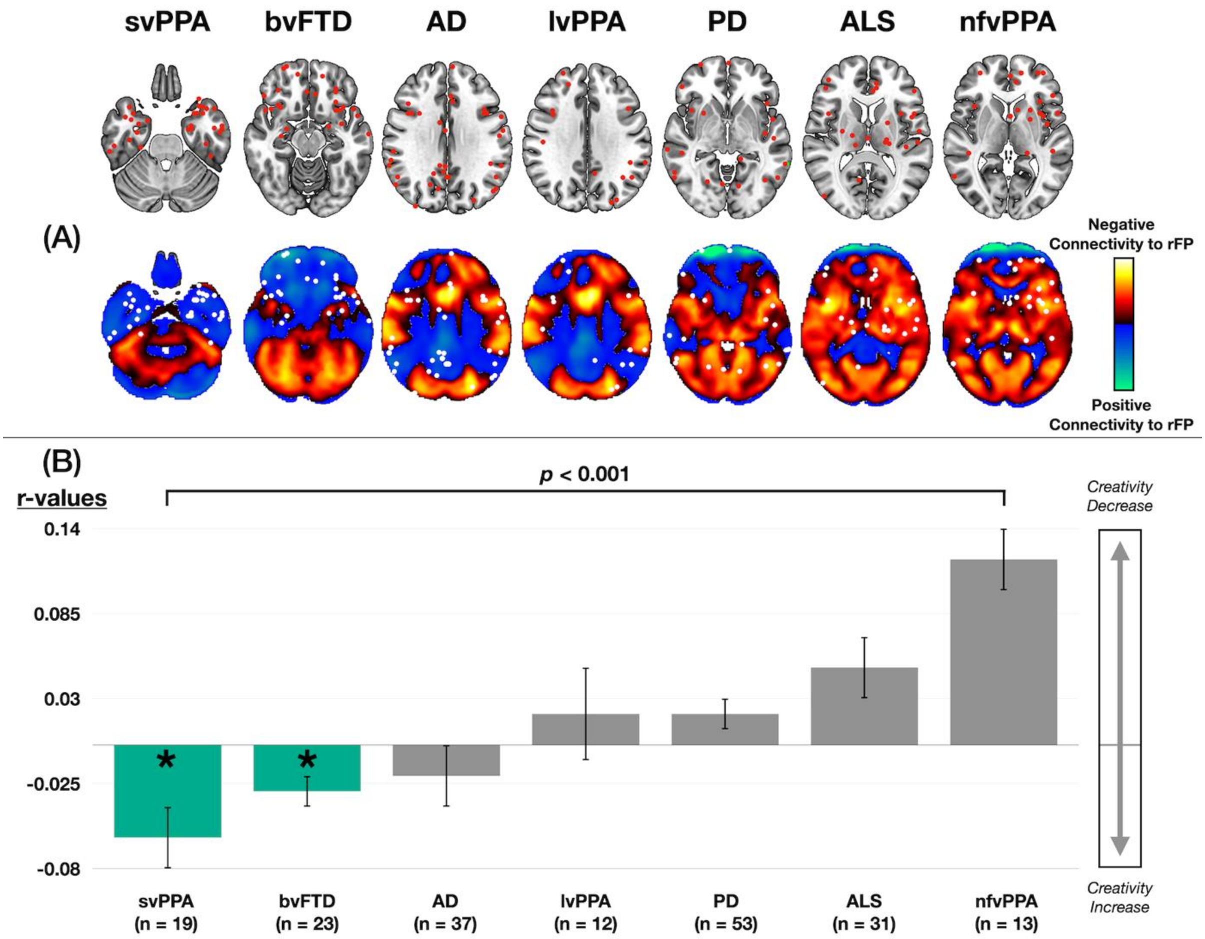

© 2025 Kutsche J et al. *JAMA Network Open*.

eFigure 6.
Scatter plot displaying the bell curve for average ROI-to-ROI correlations between 36 creativity seeds and traced atrophy for FTD patients displaying emergence of visual artistic creativity (orange) and FTD patients not displaying emergence of visual artistic creativity (blue); Atrophy sites can be found in Friedberg et al. (2023)

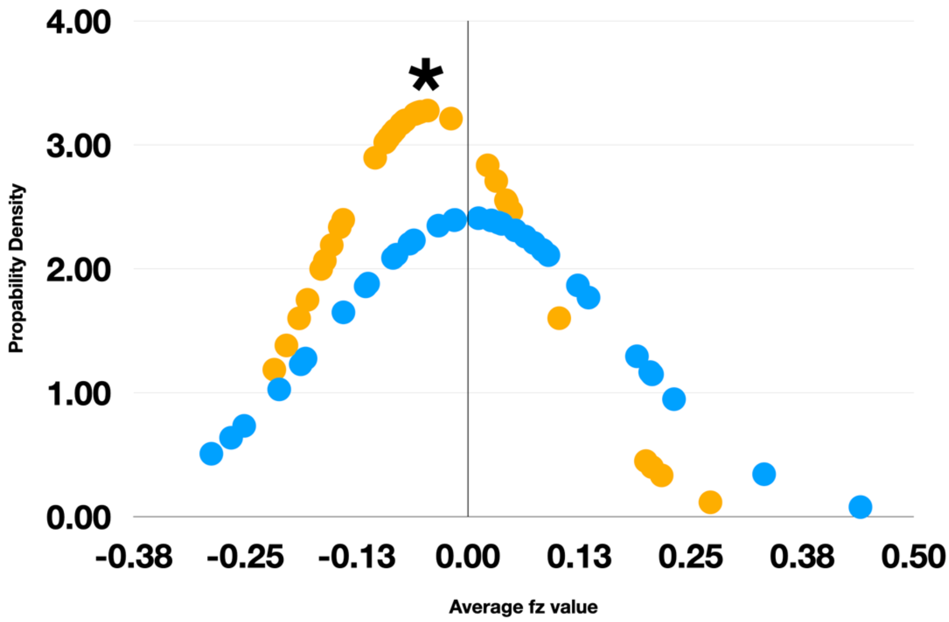

© 2025 Kutsche J et al. *JAMA Network Open*.

eFigure 7.
Network overlap computed for motoric creativity and divergent thinking separately. In both cases, a network overlap peak can be found in the frontal pole.

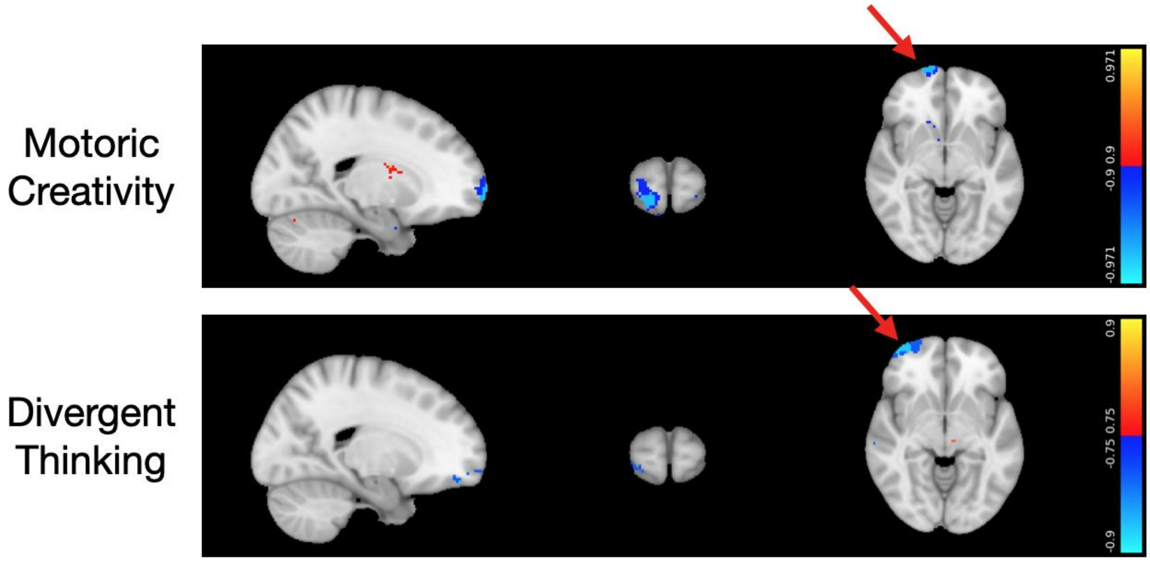

© 2025 Kutsche J et al. *JAMA Network Open*.

eFigure 8.
PRISMA diagram replicating ALE meta-analysis conducted by Brown & Kim (2021), doi:10.1515/psych-2020-0114.

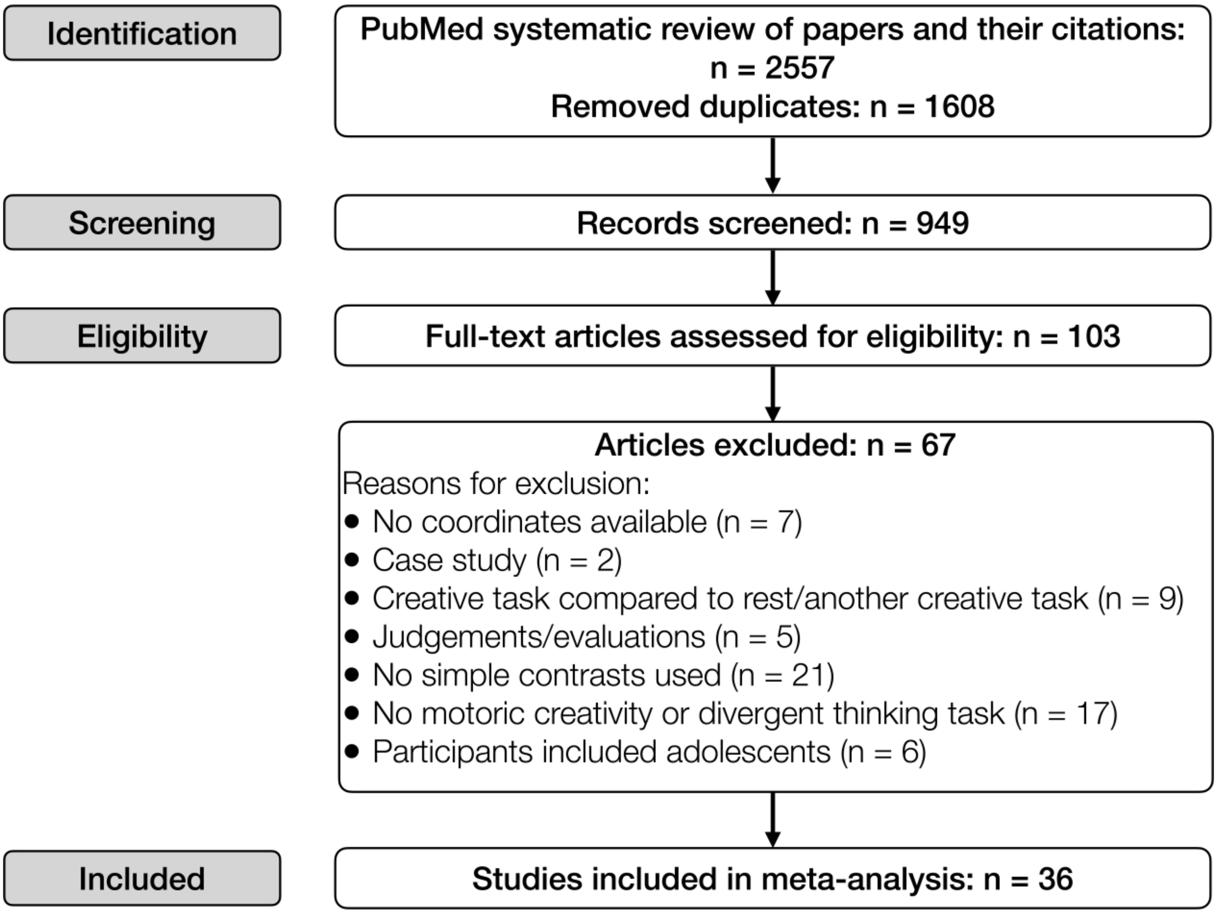

© 2025 Kutsche J et al. *JAMA Network Open*.
