# Supplementary material for: Mapping Neuroimaging Findings of Creativity and Brain Disease Onto a Common Brain Circuit
Source: JAMA Netw Open. 2025 Feb 13;8(2):e2459297. doi: 10.1001/jamanetworkopen.2024.59297 (PMC11826368; doi:10.1001/jamanetworkopen.2024.59297)
Supplement: Supplement 2. — Data Sharing Statement [file jamanetwopen-e2459297-s002.pdf]

Data Sharing Statement

Kutsche. Mapping Neuroimaging Findings of Creativity and Brain Disease Onto a Common Brain Circuit. *JAMA Netw Open*. Published February 13, 2025.
doi:10.1001/jamanetworkopen.2024.59297

Data
Data available: No

Additional Information
Explanation for why data not available: All of the data used for our analyses are already publicly available. All custom code used in this study is available upon reasonable request from the corresponding author.
